# Long non-coding RNA FAM83H-AS1 is regulated by human papillomavirus 16 E6 independently of p53 in cervical cancer cells

**DOI:** 10.1038/s41598-019-40094-8

**Published:** 2019-03-06

**Authors:** Jamie A. Barr, Karen E. Hayes, Tayvia Brownmiller, Abby D. Harold, Rajaganapathi Jagannathan, Paul R. Lockman, Saleem Khan, Ivan Martinez

**Affiliations:** 1 Department of Microbiology, Immunology & Cell Biology, West Virginia University Cancer Institute, School of Medicine, West Virginia University, Morgantown, West Virginia USA; 2 Department of Basic Pharmaceutical Sciences, Health Sciences Center, School of Pharmacy, West Virginia University, Morgantown, West Virginia USA; 3 Department of Microbiology and Molecular Genetics, School of Medicine, University of Pittsburgh, Pittsburgh, Pennsylvania USA

## Abstract

High-risk human papillomavirus (HPV) infection is one of the first events in the process of carcinogenesis in cervical and head and neck cancers. The expression of the viral oncoproteins E6 and E7 are essential in this process by inactivating the tumor suppressor proteins p53 and Rb, respectively, in addition to their interactions with other host proteins. Non-coding RNAs, such as long non-coding RNAs (lncRNAs) have been found to be dysregulated in several cancers, suggesting an important role in tumorigenesis. In order to identify host lncRNAs affected by HPV infection, we expressed the high-risk HPV-16 E6 oncoprotein in primary human keratinocytes and measured the global lncRNA expression profile by high-throughput sequencing (RNA-seq). We found several host lncRNAs differentially expressed by E6 including GAS5, H19, and FAM83H-AS1. Interestingly, FAM83H-AS1 was found overexpressed in HPV-16 positive cervical cancer cell lines in an HPV-16 E6-dependent manner but independently of p53 regulation. Furthermore, FAM83H-AS1 was found to be regulated through the E6-p300 pathway. Knockdown of FAM83H-AS1 by siRNAs decreased cellular proliferation, migration and increased apoptosis. FAM83H-AS1 was also found to be altered in human cervical cancer tissues and high expression of this lncRNA was associated with worse overall survival, suggesting an important role in cervical carcinogenesis.

## Introduction

High-risk HPV infection (e.g. HPV-16) is one of the most common causes of cervical cancer^1–3^, as well as a subset of head and neck squamous cell carcinoma (HNSCC)^1^. The HPV oncoproteins E6 and E7 have been shown to contribute to carcinogenesis by modulating the degradation of human proteins, such as the tumor suppressors p53^4^ and Rb^5^ as well as a plethora of other cellular proteins^2,3,6–8^. The HPV-16 E6 protein can abrogate p53 function by proteasomal degradation as it forms a complex with E6-associated protein (E6AP)^9^, or by targeting the p53 coactivator CBP-p300^8,10^. Upon transmission, HPV infects the undifferentiated keratinocytes at the basal layer of the stratified epithelia and its genome remains episomal maintaining low copy numbers. During the course of cancer development, the viral genome frequently becomes integrated into the host cell DNA^11^.

The recent discovery of different classes of non-coding RNAs (ncRNAs) expressed in human cells has opened a new chapter in the understanding of cellular processes, such as chromatin remodeling, transcriptional control, and post-transcriptional regulation. One of these classes of ncRNAs called long non-coding RNAs (lncRNAs) are defined as RNAs larger than 200 nucleotides that are not translated into proteins. Recent findings indicate that lncRNAs are involved in gene regulation at the transcriptional level by functioning as signal, guide, decoy, or scaffold RNAs^12–14^. Dysregulation of lncRNAs occurs in a variety of cancers, suggesting a potential use of these ncRNAs as biomarkers for diagnosis, prognosis, stage of cancer, and response to therapy^15–18^. LncRNAs have been shown to be altered in cervical cancer^19–21^, however, only a few publications have studied lncRNAs that are specifically regulated by the HPV E6 oncoprotein, such as MALAT1 and CCEPR^22,23^. These lncRNAs were found altered in cervical cancer, but it is unknown if these alterations are part of the early events in cervical carcinogenesis. A few studies have looked at aberrant expression of lncRNAs in progression from pre-malignant cervical intraepithelial neoplasia (CIN) to cervical cancer^24,25^. For these reasons, it is important to understand if certain lncRNAs are important in the first stages of immortalization and transformation caused by HPV infections.

In this study, we demonstrated that the lncRNA FAM83H-AS1 (also known as onco-lncRNA-3) is up-regulated in primary keratinocytes expressing HPV-16 oncogene E6 as well as HPV-16 positive human cervical cancer cell lines and cervical tumor samples. We show that FAM83H-AS1 is regulated by HPV-16 E6 through the presence of p300 instead of the tumor suppressor p53. Finally, we show that FAM83H-AS1 is involved with cellular proliferation, migration, and apoptosis and is associated with worse overall survival in cervical cancer patients.

## Results

### High-risk HPV-16 E6 oncoprotein alters host long non-coding RNAs in primary keratinocytes

**Figure 1:**
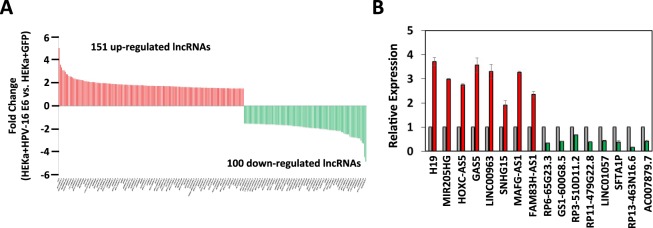
Differential expression of host lncRNAs after expression of HPV-16 E6 in primary foreskin keratinocytes. (**A**) Waterfall plot of host lncRNAs altered 1.5-fold or greater in primary foreskin keratinocytes (HEKa) expressing HPV-16 E6 compared to HEKa expressing GFP by high-throughput RNA sequencing (RNA-seq) analysis. Triplicates for each sample were sent for RNA-seq. (**B**) qRT-PCR validation of representative differentially expressed host lncRNAs found by RNA-seq analysis. Red bars represent the lncRNAs up-regulated with HPV-16 E6 expression, and green bars represent the lncRNAs down-regulated with HPV-16 E6 expression. GAPDH mRNA was used to normalize the qRT-PCR analyses, which are shown relative to uninfected HEKa (grey bars).

As an initial screen to identify host lncRNAs that are regulated specifically by HPV-16 E6, we developed a system to look specifically at the effect of E6 expression alone in primary human foreskin keratinocytes (HEKa). HEKa were infected with a retroviral vector expressing HPV-16 E6 oncogene or GFP as a control. After puromycin selection and stable expression of HPV-16 E6 was confirmed by RT-PCR (Fig. S1), RNA was extracted, and samples were analyzed by RNA high-throughput sequencing (RNA-seq) to determine gene expression alterations in long non-coding RNAs (lncRNAs). Following bioinformatics analysis, we found 151 up- and 100 down-regulated host lncRNAs altered greater than 1.5-fold change when HPV-16 E6 was expressed in HEKa cells compared to GFP control (Fig. 1A, Table S1). From these host lncRNAs, we randomly chose 8 up- and 8 down-regulated host lncRNAs to validate our RNA-seq data by qRT-PCR. The expression of all the lncRNAs selected for validation followed the same trend of up- or down-regulation found by RNA-seq (Fig. 1B). In order to determine the importance of these 251 lncRNAs in cervical carcinogenesis, a variety of filtering methods were utilized to reduce our scope (see Methods section). After filtering, we performed preliminary experiments with many lncRNAs, however, some of our top altered lncRNAs from our RNAseq analysis of foreskin keratinocytes (HEKa) were not altered in cervical cells (HCK) with HPV-16 E6 expression (e.g. SNHG15). This is not surprising, as lncRNAs are known to typically be tissue specific^26^. We also found differences in the expression of some lncRNAs between pre-malignant and cancerous cervical cells CaSki and W12/201402 to HCK (e.g. miR205HG). One of the up-regulated lncRNAs in our RNA-seq dataset, FAM83H-AS1 was intriguing. Its expression recently was found to correlate with poor overall survival in a variety of human cancers^27–32^ but had not previously been shown in the context of cervical cancer. In addition, it was recently shown to be involved in regulating cellular processes associated with the hallmarks of cancer (e.g. proliferation and migration)^28,31,32^. To ensure that expression of FAM83H-AS1 is comparable to lncRNAs with well-known functions, we utilized our RNA-seq dataset to compare their RPKM values (Table S2).

### FAM83H-AS1 expression is higher in HPV-16+ pre-malignant and cancerous samples

**Figure 2:**
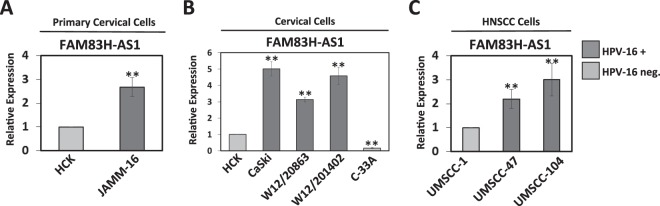
Increased FAM83H-AS1 expression in primary cervical keratinocytes containing the HPV-16 genome as well as in HPV-16 positive cervical cancer and head and neck squamous cell carcinoma cell lines. (**A**) qRT-PCR analysis showing the increase of FAM83H-AS1 expression in newly immortalized cervical keratinocytes expressing entire HPV-16 genome (JAMM-16) compared to uninfected primary cervical keratinocytes (HCK). (**B**) qRT-PCR analysis showing the increase of FAM83H-AS1 expression in HPV-16 positive cervical cell lines (CaSki, W12/20863, W12/201402) and decrease of FAM83H-AS1 in HPV negative cervical cancer cell line (C-33A) compared to uninfected cervical keratinocytes (HCK). (**C**) qRT-PCR analysis showing the increase of FAM83H-AS1 expression in HPV-16 positive head and neck squamous cell carcinoma (HNSCC) cell lines (UMSCC-47 and UMSCC-104) compared to HPV negative HNSCC cell line (UMSCC-1). All graphs in the figure show the average of two individual experiments. Similar results were obtained in at least three independent experiments. GAPDH mRNA was used to normalize the qRT-PCR analyses. Two-tailed t test results are indicted as **p ≤ 0.01.

Because many lncRNAs are known to be tissue specific^26^ and our RNA-seq screening was performed with foreskin keratinocytes expressing HPV-16 E6 oncogene, it was critical to confirm the expression changes of FAM83H-AS1 in epithelial keratinocytes of the cervix where HPV naturally infects^11^. Additionally, we considered that the E6 and E7 oncogenes can be synergistic with each other^33^, so we needed to develop a model that closely mimics HPV infection by using the entire HPV-16 genome. Therefore, primary human cervical keratinocytes (HCK) were transfected with the entire HPV-16 genome (by releasing the viral genome from a plasmid construct and circularizing it by ligation before transfection), then passaged several times (around 10–15 divisions) for growth selection of HPV positive immortalized cells. HPV-16 E6 and E7 expression was confirmed by RT-PCR (Fig. S2A), as well as p53 degradation through the HPV-16 E6 pathway by Western Blot (Fig. S2B). After confirmation of HPV-16 oncogene expression in these cells, we named them JAMM-16. It was confirmed by qRT-PCR that FAM83H-AS1 is also up-regulated in JAMM-16 cervical keratinocytes expressing the entire HPV-16 genome in comparison to the parental cervical keratinocytes (Fig. 2A). We then used HPV-16 positive low-grade cervical (W12/20863 [episomal HPV-16], W12/201402 [integrated HPV-16]) and carcinoma (CaSki [integrated HPV-16]) cell lines to investigate the expression of FAM83H-AS1. As shown in Fig. 2B, we found higher expression of FAM83H-AS1 in all the HPV-16 positive cell lines in comparison to HCK cells. Interestingly, FAM83H-AS1 was expressed at lower levels in HPV-negative cervical cancer C33A cells compared to HCK as well as HPV-16 positive cervical cells (Fig. 2B). Furthermore, HPV-16 positive and HPV-negative head and neck squamous cell carcinoma (HNSCC) cell lines were compared, and higher expression of FAM83H-AS1 was observed in the HPV-16 positive versus the HPV-negative HNSCC (Fig. 2C). Overall, we conclude that presence of HPV-16 correlates with elevated levels of FAM83H-AS1 expression in early stages of cervical carcinogenesis (newly immortalized JAMM-16 cells and cervical low-grade pre-malignant cell lines) as well as cervical cancer and HNSCC cell lines.

### FAM83H-AS1 expression is regulated by HPV-16 E6 in a p53-independent, p300-dependent manner

**Figure 3:**
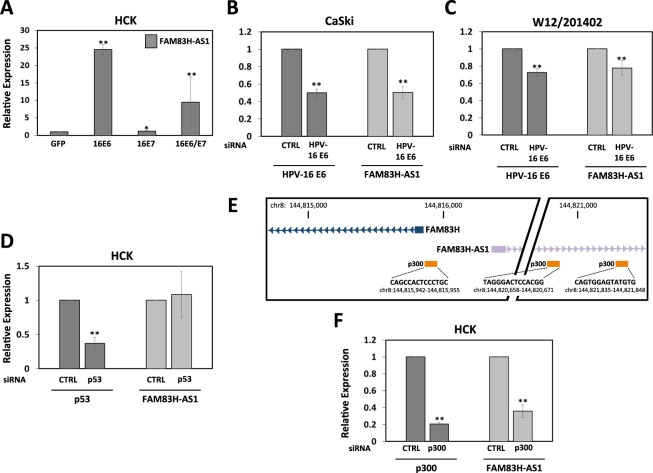
Regulation of FAM83H-AS1 expression by HPV-16 E6 in a p53-independent, p300-dependent manner. (**A**) FAM83H-AS1 expression by qRT-PCR analysis in cervical keratinocytes (HCK) stably individually expressing HPV-16 E6 or E7, or co-expressing E6/E7 compared to GFP control. (**B**–**C**) qRT-PCR analysis of HPV-16 E6 and FAM83H-AS1 expression in HPV-16 positive CaSki (**B**) and W12/201402 (**C**) cervical cell lines transfected with an siRNA against HPV-16E6 compared to siRNA control. (**D**) qRT-PCR analysis of p53 and FAM83H-AS1 expression in HCK transfected with an siRNA against p53 compared to siRNA control. (**E**) Genome representative image showing location of FAM83H, FAM83H-AS1, and three predictive p300 binding sites in FAM83H-AS1 promoter region. (**F**) p300 and FAM83H-AS1 expression in HCK transfected with an siRNA against p300 compared to siRNA control. All graphs in the figure show the average of two individual experiments. Similar results were obtained in at least three independent experiments. GAPDH mRNA was used to normalize the qRT-PCR analyses. Two-tailed t test results are indicted as *p ≤ 0.05 and **p ≤ 0.01. CTRL, control.

As shown in Fig. 1, foreskin keratinocytes (HEKa) expressing HPV-16 E6 up-regulated FAM83H-AS1 expression. To further confirm HPV-16 E6 regulation of FAM83H-AS1 in cervical cells, cervical keratinocytes (HCK) stably expressing HPV-16 E6 were developed (Fig. S3). To measure if FAM83H-AS1 regulation could be affected by HPV-16 E7 oncogene in synergistic or antagonistic manner, we generated HCK stable cell lines expressing HPV-16 E7 or co-expressing HPV-16 E6 and E7 (Fig. S3). FAM83H-AS1 was up-regulated when cells expressed HPV-16 E6 or HPV-16 E6 and E7, but not when they expressed HPV-16 E7 alone (Fig. 3A) suggesting a specific regulation by E6. To further confirm these findings, HPV-16 E6 was knocked down in CaSki (Figs 3B and S4A) and W12/201402 (Figs 3C and S4B) cell lines by two different siRNAs against HPV-16 E6-E7. FAM83H-AS1 expression was down-regulated after the reduction of HPV-16 E6 expression confirming the regulation of this lncRNA by HPV-16 E6. It is well known that one of the major HPV E6 targets is the tumor suppressor p53^34^, which is involved in the regulation of cell proliferation, DNA repair, and apoptosis^35^. Interestingly, when we knocked down p53 in HCK by using two different siRNAs, we observed that FAM83H-AS1 expression did not change (Figs 3D and S4C), suggesting a regulation by HPV-16 E6 in a p53-independent manner. It is also known that HPV-16 E6 is able to regulate the expression of other important genes in carcinogenesis such as hTERT through the regulation of transcriptional coactivators such as p300^36^. By using the UCSC Genome Browser (https://genome.ucsc.edu/) to investigate potential p300 binding site in the promoter region of FAM83H-AS1, we found three predicted p300 binding sites (Fig. 3E). In order to measure the potential regulation of FAM83H-AS1 by p300, we used two siRNAs against p300 and quantified FAM83H-AS1 expression. When p300 was knocked down in primary cervical keratinocytes, FAM83H-AS1 expression was also reduced (Figs 3F and S4D), suggesting direct and/or indirect p300 regulation of FAM83H-AS1. Previous publications have shown a greater affinity of HPV-16 E6 to interact with p300 in comparison with other high-risk HPV E6 such as HPV-18 E6^37^. Interestingly, FAM83H-AS1 expression was found to be up-regulated in HPV-16 positive cell lines (Fig. 2), but down-regulated in HPV-18 positive HeLa cells and HPV-31b positive CIN-612 cells (Fig. S4E). Altogether, we found that FAM83H-AS1 is regulated by HPV-16 E6 independently of p53 but influenced by the presence of p300.

### FAM83H-AS1 is localized to the nucleus and does not regulate transcription of nearby FAM83H

**Figure 4:**
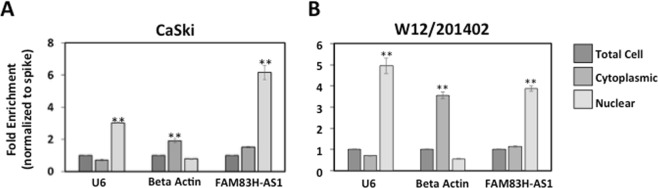
FAM83H-AS1 is localized in the nucleus in cervical pre-malignant and cancerous cell lines. (**A**) qRT-PCR of FAM83H-AS1 expression in fractionated HPV-16 positive cervical cancer CaSki (**A**) and pre-malignant W12/201402 (**B**) cell lines. U6 small nuclear RNA (snRNA) was used as a nuclear control RNA and mature Beta Actin was used as a cytoplasmic control RNA. Representative images; similar results were obtained in at least three independent experiments. Normalization was done using *C*. *Elegans* total RNA as an exogenous spike for the amplification of worm-specific *ama-1* gene. Two-tailed t test results are indicted as **p ≤ 0.01.

The cellular localization of lncRNAs can provide information on their potential function. Nuclear lncRNAs can regulate at the transcriptional level by interacting with critical epigenetic regulators and enhancing chromatin looping, as well as interact with splicing factors to regulate splicing^38^. Meanwhile cytoplasmic lncRNAs have been found to function at the post-transcriptional level as competing endogenous RNAs by acting as microRNA sponges and binding to mRNAs leading to the recruitment of RNA binding proteins that promote decay, suppress translation, or factors that initiate translation^39^. For this reason, we investigated the cellular localization of FAM83H-AS1 in two HPV-16 positive cervical cell lines by cellular fractionation. We used U6 as a nuclear RNA control and β-actin mature mRNA as a cytoplasmic RNA control. We found significant enrichment of FAM83H-AS1 in the nuclear fractions in comparison to the cytoplasmic fractions by qRT-PCR in CaSki (Fig. 4A) and W12/201402 (Fig. 4B) cells, suggesting that FAM83H-AS1 is a nuclear lncRNA. Because many nuclear lncRNAs can act in cis^38^, we hypothesized that FAM83H-AS1 could regulate its nearby protein coding gene FAM83H. The lncRNA FAM83H-AS1 and protein coding gene FAM83H share a promoter region but are transcribed in opposite directions^27^ (Figs 3E and S5). FAM83H is required for the organization of the keratin cytoskeleton in epithelial cells^40^ and has been shown over-expressed in different tumor samples compared to their matching normal tissues^41^. Interestingly, we found increased expression of FAM83H in HCK expressing HPV-16 E6 in comparison to parental HCK cells (Fig. S5B), but when we transfected an siRNA against FAM83H-AS1 in HPV-16 positive CaSki cells, we were unable to detect changes in FAM83H expression (Fig. S5C), suggesting that FAM83H-AS1 is not involved in regulation of FAM83H expression.

### FAM83H-AS1 knockdown in cervical cancer cells causes reduced cellular proliferation and migration, as well as induction of apoptosis

**Figure 5:**
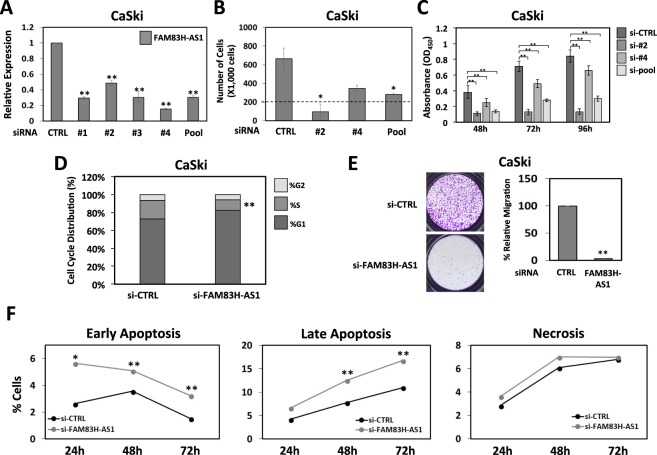
FAM83H-AS1 knockdown altered cell proliferation, migration, and apoptosis in CaSki cells. (**A**) Knockdown efficiency of individual and SMARTpool siRNA against FAM83H-AS1 in HPV-16 positive CaSki cell line, measured by qRT-PCR analysis. Because of variations in the expression of GAPDH after the knockdown of FAM83H-AS1, we used UBC mRNA to normalize the qRT-PCR analyses. The graph shows average of two individual experiments. (**B**) CaSki cells were transfected with individual siRNAs against FAM83H-AS1, siRNA SMARTpool against FAM83H-AS1, or siRNA control for 48 hours. Cells were then re-plated in equal numbers (200,000 cells/well, represented by dashed line in graph) and cultured another 48 hours prior to re-counting attached cells. Data were obtained in triplicate, and the graph shows the average of two individual experiments. (**C**) CaSki cells were transfected with individual siRNAs against FAM83H-AS1, siRNA SMARTpool against FAM83H-AS1, or siRNA control for 24 hours then plated in equal numbers. Transfected cells were analyzed for cellular proliferation assessment by CCK-8 assay at 48, 72, and 96 hours post-plating. The graph shows the average of two individual experiments; similar results were obtained in three independent experiments. (**D**–**F**) CaSki cells were transfected with siRNA SMARTpool against FAM83H-AS1 or siRNA control for 24 hours then plated in equal numbers for experiments. (**D**) Transfected cells were analyzed for cell cycle alterations by FACS analysis. CaSki cells with knockdown of FAM83H-AS1 exhibit less cells in S-phase of cell cycle compared to control cells. The graph shows the average of two individual experiments; similar results were obtained in three independent experiments. (**E**) Transwell migration of transfected cells was analyzed 48 hours post-plating in upper chamber with chemoattractant in lower chamber. The graph shows the average of three individual experiments. (**F**) Transfected CaSki cells were collected at 1, 2, and 3 days post-plating, stained with Annexin V/PI, and analyzed by flow cytometry to show alterations in apoptosis compared to siRNA control. The graph shows the average of three individual experiments. Two-tailed t test results are indicted as *p ≤ 0.05 and **p ≤ 0.01.

To understand the significance of FAM83H-AS1 in cervical cancer cells, we analyzed the effects on cellular proliferation when FAM83H-AS1 was knocked down by siRNA in CaSki and W12/201402 cervical cells. First, knockdown efficiency of a pool of 4 individual siRNAs (SMARTpool), as well as each of the individual siRNAs, was evaluated by qRT-PCR. Knockdown of FAM83H-AS1 in CaSki with the siRNA SMARTpool was maintained over a time-course from 24 to 120 hours, which was sufficient for all functional assays conducted (Fig. S6). All of the individual siRNAs, as well as the SMARTpool showed greater than 51% knockdown of FAM83H-AS1 in CaSki (Fig. 5A) and greater than 49% in W12/201402 cells (Fig. S7A). Therefore, we decided to randomly choose two of the individual siRNAs and SMARTpool to knockdown FAM83H-AS1 and monitor cellular functional changes. Two siRNAs against FAM83H-AS1 were transfected independently into CaSki and W12/201402 cells, cultured for 48 hours, and replated to measure cell proliferation by cell counting. In both CaSki and W12/201402 cells, we observed a decrease (≥48%) in cell number with knockdown of FAM83H-AS1 compared to control (Figs 5B and S7B). Cellular proliferation assay (CCK-8) showed that there was a decrease in cellular proliferation when FAM83H-AS1 is knocked down in CaSki cells and W12/201402 as monitored from 48 hours to 96 hours after replating. We found significantly less proliferation in CaSki (64% decrease) and W12/201402 (73% decrease), in the siRNA FAM83H-AS1 knockdown compared to siRNA control cells at the 96-hour time point (Figs 5C and S7C). We observed similar functional changes between the two individual siRNAs and the SMARTpool so for future functional assays we only used the siRNA SMARTpool. In order to identify changes in cell cycle that could explain the differences found in cellular proliferation after FAM83H-AS1 knockdown, we performed cell cycle flow cytometry analysis. CaSki and W12/201402 cells had a significant reduction (43% and 56%, respectively) of cells in S-phase when FAM83H-AS1 was knocked down in comparison to control suggesting that FAM83H-AS1 is important in the G2/S-phase transition (Figs 5D and S7D). Other important hallmarks of cancer such as migration and resistance to apoptosis were measured after FAM83H-AS1 knockdown. We found that cellular migration was significantly decreased in CaSki and W12/201402 after siRNA knockdown of FAM83H-AS1 compared to siRNA control (Figs 5E and S7E). Also, we measured a significant increase in early and late apoptosis in CaSki and W12/201402 cells with knockdown of FAM83H-AS1 compared to control cells by using Annexin V/PI staining and flow cytometry (Figs 5F and S7F-H). W12/201402 cells with knockdown of FAM83H-AS1 showed a significant increase in necrosis (Fig. S7F), while only a slight increase in necrosis was observed in CaSki cells (Figs 5F and S7H). Altogether, we observed significant alterations in cellular proliferation, cell cycle, migration, and apoptosis by the absence of FAM83H-AS1, suggesting an important role in cervical carcinogenesis.

### FAM83H-AS1 expression is increased in cervical cancer human tissues & is associated with worse overall survival

**Figure 6:**
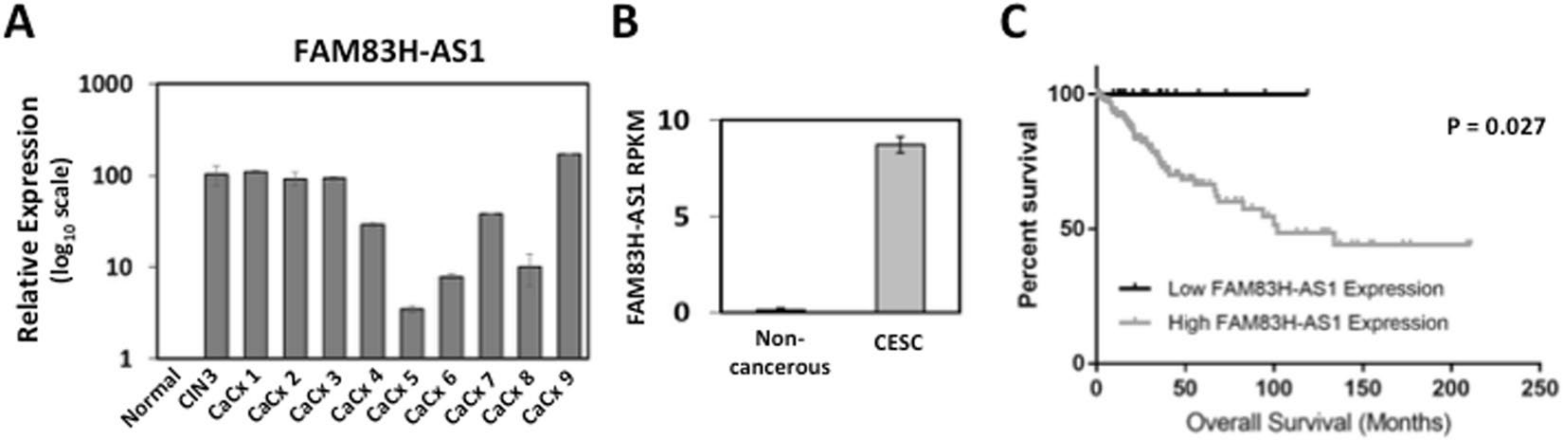
FAM83H-AS1 expression is increased in human cervical cancer tissues and correlates with poor overall survival. (**A**) Increased FAM83H-AS1 expression in human cervical cancer and cervical intraepithelial neoplasia (CIN) stage 3 patient samples compared to non-cancerous cervical tissue as measured by qRT-PCR analysis. (**B**) The Cancer Genome Atlas (TCGA) analysis of FAM83H-AS1 RPKM values in cervical cancer tissues (n = 196) compared to non-cancerous tissues (n = 3). (**C**) Survival plot of cervical squamous cell carcinoma and endocervical adenocarcinoma (CESC) patient data mined from TCGA with low (n = 22) vs. high (n = 174) expression of FAM83H-AS1. High expression of FAM83H-AS1 expression correlates with worse overall survival in CESC patients.

In order to extrapolate our findings into a more clinically relevant setting, we extracted RNA from pre-malignant and cervical cancer patient samples and analyzed the expression of FAM83H-AS1 by qRT-PCR. We found high expression of FAM83H-AS1 in the pre-malignant sample (CIN3) as well as the cervical cancer (CaCx) samples in comparison to normal cervix tissue (Fig. 6A). These findings corroborate our *in vitro* data suggesting an importance of FAM83H-AS1 in clinical tumor samples at different stages of carcinogenesis. We also took advantage of the cervical cancer samples deposit in the TCGA database to compare the expression of FAM83H-AS1 between normal cervix and cervical cancer samples obtained from different cancer stages (120 Stage I, 35 Stage II, 30 Stage III, 7 Stage IV, 4 Stage unavailable). The TCGA data showed elevated expression of FAM83H-AS1 (RPKM values) in cervical cancer patients compared to normal cervix control (Fig. 6B). This coincides with our previous observations of FAM83H-AS1 expression being higher in cervical cancer cells lines (Fig. 2B). Finally, we used the TCGA data set from the TANRIC database to divide cervical cancer patients into high versus low expression groups and measured overall survival based on FAM83H-AS1 expression. Interestingly, we found that patients with higher FAM83H-AS1 expression yielded a worse overall survival than patients with lower FAM83H-AS1 expression suggesting a biological importance of this lncRNA in cervical cancer reflected in patients’ clinical outcomes (Fig. 6C). Overall, we conclude that FAM83H-AS1 expression is elevated in cervical cancer patients and high expression correlates with overall poor survival.

## Discussion

4.5% of all cancers worldwide are attributable to HPV infection. Almost all cervical cancers and a substantial amount of other anogenital and oropharyngeal cancers have been found to be infected by high-risk HPVs. HPV-16 and -18 contribute to 73% of HPV-associated cancers^42^, implying a higher ability to induce tumorigenesis in comparison to other types of HPVs. Although the prevalence of HPV-associated cancers has decreased due to development of the preventative vaccine and early detection screening methods^2^, there is still a great need for prognostic and therapeutic options specially to people already infected with HPV as well as those affected in less developed countries where the access to the vaccine is limited.

Long noncoding RNAS (lncRNAs) have been shown to regulate a variety of critical cellular processes, including transcription and chromosome remodeling^12–14^. Dysregulation of lncRNAs has been shown to be associated with the development and progression of many cancers^15–17^, and interestingly they are commonly tissue specific^26^ and only altered in one cancer type^32^. Therefore, lncRNAs are currently being studied in the context of biomarkers for diagnosis and prognosis of cancer, as well as therapeutic targets^15–18^.

Previous studies have shown that the high-risk HPV E6 protein expressed is clearly involved in the progression of carcinogenesis. HPV E6-regulation of non-coding RNAs such as microRNAs has been well studied^43^, however long non-coding RNA regulation by high-risk HPV E6 needs to be studied further; there are only a couple lncRNAs shown to be specifically HPV-16 E6 regulated, including MALAT1 and CCEPR^22,23^. To add to this field of study, we sought out to identify an HPV-16 E6 regulated gene that was altered from the early stages of HPV infection until carcinogenesis and therefore we considered to be important in both the development and progression of carcinogenesis. Thus, we developed a new HPV-16 positive cell line referred to as JAMM-16 to represent early infection, but also analyzed expression in established HPV-16 positive cervical cell lines such as CaSki and W12 cells as well as pre-malignant and malignant cervical tumor samples. We found FAM83H-AS1 overexpression in W12/20863 and W12/201402 (which came from a CIN2 tumor) similar to CaSki cells (cervical carcinoma). Additionally, in Fig. 6A, the CIN3 (considered Stage 0 cervical carcinoma) patient sample shows similar expression to later stage cervical carcinoma (CaCx) samples. For this reason, we believe that FAM83H-AS1 up-regulation in pre-malignant cervical samples could be linked to the expression of viral oncogenes in early HPV infection.

From the host lncRNAs altered by HPV-16 E6 in our RNA-seq analysis (Fig. 1), we found several of these lncRNAs previously described to be altered in cervical cancers, confirming that our data aligned with former studies. For example, it was previously found that decreased expression of GAS5 is associated with poor prognosis of cervical cancer patients^44,45^ as well as is tumor suppressive in other types of cancer such as breast cancer^46,47^ and prostate cancer^47^. Furthermore, GAS5 expression was also found altered *in vitro* in HPV-16 positive CaSki cells^44^ and HPV-18 positive HeLa cells^45^. Another lncRNAs affected in our study was H19. It was shown previously that DNA methylation alterations at the IGF2/H19 imprinted domain may mediate the association between HPV and invasive cervical cancer^48^ and high H19 expression has also been shown to be predictive of poor prognosis in cervical cancer^49^ as well as in a variety of other human cancers, including HNSCC^50^ and breast cancer^51^.

A class of lncRNAs known as onco-lncRNAs were also interesting to us because, as the majority of lncRNAs are tissue specific^26^, this group of lncRNAs exhibit differential expression across multiple cancers and are hypothesized to have conserved oncogenic or tumor suppressive functions. One such onco-lncRNA which was found in our RNA sequencing analysis of an E6-regulated lncRNA is onco-lncRNA-3, referred to as FAM83H-AS1. This lncRNA is transcribed from chromosome 8 and its function in normal cells is unknown. It was first characterized in 2015^32^, and as of now multiple publications have shown increased expression of FAM83H-AS1 in breast^29,32^, lung^31,32^, colorectal^28,30,32^, kidney^32^, bladder^32^, and pancreatic cancers^27^ and increased expression correlates with worse overall survival in most of these cancers^27,29–31^. Our findings show for the first time that FAM83H-AS1 is overexpressed in human cervical cancer (CESC) tissues and high expression in patients correlates with poor overall survival (Fig. 6).

According to previous studies, FAM83H-AS1 is an epithelial lncRNA^27^ supporting our data obtained from foreskin and cervical keratinocytes. Determining the localization of a lncRNA can predict functionality of the lncRNA; our findings that FAM83H-AS1 is localized in the nucleus of cervical cancer cells (Fig. 4) is consistent with previous findings of its nuclear localization in lung cancer cells^14^. Functionally, it has been found to be co-expressed with protein coding genes that were enriched for cell cycle-related genes^32^, and knockdown of FAM83H-AS1 altered cell cycle^31,32^, proliferation^28,31^, migration^28,31^, invasion^31^, and apoptosis^28^ in certain cancers. Our group shows here that in the context of cervical cancer, FAM83H-AS1 is involved in cell cycle, proliferation, migration, and apoptosis (Fig. 5). It is unknown if FAM83H-AS1 elicits its functions in cis or trans, but our findings suggest that FAM83H-AS1 does not elicit cis regulation on the nearby protein coding gene FAM83H (Fig. S5), which is up-regulated in a variety of human cancers. For this reason, it will be interesting in future studies to identify the protein, RNA, and/or DNA interactions of FAM83H-AS1 in cervical cancers.

Previously, it was shown that FAM83H-AS1 regulates MET/EGFR signaling in lung cancer cells^31^ and that when FAM83H-AS1 was downregulated it exhibited an anti-proliferative role by suppressing the Notch signaling pathway in colorectal cancer^28^. To elucidate additional downstream targets of FAM83H-AS1, a group recently conducted RNA-seq on a pancreatic cancer cell line with siRNA knockdown of FAM83H-AS1 compared to control and identified gene alterations (78 activated and 68 inhibited targets)^27^. Our group plans to determine if these downstream regulators are also involved in FAM83H-AS1 mediated functional changes observed in cervical cancer cells.

HPV-16 E6 and -18 E6 are well known to contribute to the degradation of p53, however, it is important to note that HPV-16 and HPV-18 vary in their interactions with other proteins to regulate carcinogenesis. For example, previous studies have shown that HPV-16 E6 directly interacts with CBP/p300^8,10,37^, but HPV-18 E6 appears to be unable to interact with p300^37^. This could be a possible explanation for variation in FAM83H-AS1 expression between HPV-16 and -18 positive cancers observed in our study (Figs 2 and S4E). Supporting our data, another publication previously showed low expression of FAM83H-AS1 in HeLa (HPV-18 positive) cells^27^. This information led us to elucidate the mechanism of FAM83H-AS1 up-regulation by E6 in a p53-independent and p300-dependent manner (Figs 3 and S4). This regulation is interesting because the majority of E6 regulation of several coding and non-coding genes is primarily through the p53 pathway. Interestingly, a previous study showed that overexpression of cyclooxygenase (COX-2) gene was the result of the recruitment of p300 to its promoter region via the overexpression of HPV-16 E6 protein in CaSki cells as well as the exogenous expression of HPV-16 E6 in HPV-negative C-33A cells^52^. Recently, p300 inhibitors such as C646 have been shown to be good candidates as anti-cervical cancer drugs, demonstrating the importance of p300 not only in the regulation of host genes but also of HPV viral genes^53^.

In summary, the identification of FAM83H-AS1 up-regulation in the early stages of cervical carcinogenesis, correlation with overall survival in cervical cancer, and involvement in different hallmarks of cancer contributes further evidence of the importance of this lncRNA in cancer. Further studies on this lncRNA could enhance the use of FAM83H-AS1 as a potential biomarker or therapeutic target in multiple cancers.

## Methods

Detailed experimental protocols are described in the Supplementary Methods section. All experiments were performed in compliance with the Institutional Biosafety Committee at West Virginia University, number 15-03-03.

### Cells

The following cell lines were used: human primary foreskin keratinocytes (HEKa) (Invitrogen, C-005-5C); human primary cervical keratinocytes (HCK) and J2–3T3 murine fibroblast feeder cells (obtained from Dr. Alison McBride’s laboratory, NIH, Bethesda, MD); 3T3M murine fibroblast feeder cells, as well as CaSki (HPV-16 positive), HeLa (HPV-18 positive), and C-33A (HPV negative) cervical carcinoma cells (obtained from Dr. Daniel DiMaio’s laboratory, Yale University, New Haven, CT); W12/201402 (HPV-16 positive) and W12/20863 (HPV-16 positive) pre-malignant cervical cells (obtained from Paul F. Lambert, University of Wisconsin-Madison, Madison, WI); CIN-612 (HPV-31b positive) (obtained from Dr. Laimonis A. Laimins’ laboratory, Northwestern University, Chicago, IL); UMSCC-1 (HPV negative), UMSCC-47 (HPV-16 positive), and UMSCC-104 (HPV-16 positive) head and neck squamous cell carcinoma (HNSCC) cell lines (obtained from Dr. Scott A. Weed’s laboratory, West Virginia University, Morgantown, WV). Further details are in the Supplementary Methods section.

### High-throughput RNA sequencing

Three replicates each of human foreskin keratinocytes (HEKa) stably expressing HPV-16 E6 or GFP were sent for RNA high-throughput sequencing (Illumina). FASTQ files were subsequently imported into Strand NGS suites for analysis. Reads were aligned to the human hg19 reference genomes using the Bowtie algorithm. These were then quantified against Ensemble transcript and including small and lncRNA annotations. Any lncRNA that were detected in human subjects were used for further analysis. Raw lncRNA counts were then normalized to the total number of lncRNA reads per sample and expression values calculated against the control samples. Further Mapping rate visualization done using Strand NGS software. In order to determine the importance of the lncRNAs obtained from the RNA-seq analysis, we used the following filtering strategy: First, only lncRNAs with reasonable expression (RPKM greater than 1) were analyzed further. Then, we used The Atlas of Noncoding RNAs in Cancer (TANRIC, MD Anderson Cancer Center)^54^, which contains 297 sequenced human cervical squamous cell carcinoma and endocervical adenocarcinoma (CESC) patient data from The Cancer Genome Atlas (TCGA), to analyze the expression and clinical outcomes of these lncRNAs in patient samples. Finally, we searched previous publications to identify lncRNAs altered in other types of cancer and/or involved in hallmarks of cancer. To increase our novelty, we eliminated lncRNAs that were previously shown to be involved specifically in cervical cancer (e.g. H19).

### Functional Analysis

For cell counting experiments, CaSki cells were transiently transfected with Lincode Human FAM83H-AS1 siRNA SMARTpool (Dharmacon, R-188909-00-000), Individual: Lincode FAM83H-AS1 siRNA (N-188909-02-0002 and N-188909-04-0002), or Lincode Non-targeting siRNA #1 (Dharmacon, D-001320-01-05) using Lipofectamine® RNAiMAX according to manufacturer’s instructions (Invitrogen).

CaSki cells were cultured with siRNA-containing media for 48 hours, re-plated in equal cell numbers (200,000 cells/well of 6-well), cultured for another 48 hours, and attached cells were re-counted with a hemacytometer.

For all other functional assays (CCK-8 cell proliferation, FACS cell cycle, transwell migration, annexin V-FITC/PI apoptosis), CaSki cells were transiently transfected with Lincode Human FAM83H-AS1 siRNA SMARTpool or Lincode Non-targeting siRNA #1 (Dharmacon, D-001320-01-05) using Lipofectamine® RNAiMAX according to manufacturer’s instructions (Invitrogen). Cells were incubated with siRNA-containing media for 24 hours then re-plated in equal cell numbers for to initiate experiments described below. To monitor cell proliferation, transfected cells were plated in 96-well plates and after 24, 48, 72, and 96 hours analyzed with CCK-8 kit (Sigma-Aldrich) according to manufacturer’s protocol. Alterations in cell cycle were determined by flow cytometry propidium iodide DNA staining. Transfected cells were plated in media containing 10% fetal bovine serum (FBS). Cells were allowed to attach and then were serum-starved for 24 hours. Samples were then fixed with ethanol, stained with propidium iodide, and analyzed by flow cytometry (Fortessa S10). For transwell migration assay, transfected cells were seeded onto upper chambers of transwell inserts (8 µm pore size) with 20% FBS chemoattractant in the lower chamber of 24-well plate. After 48 hours, migrated cells located on the underside of the transwell insert were stained with 0.5% crystal violet in methanol. Migrated cells were quantified using ImageJ software. To monitor apoptosis, transfected cells were plated and incubated 24, 48, and 72 hours. At desired time point, attached and floating cells were pelleted and co-stained with annexin V-FITC and propidium iodine and immediately analyzed by flow cytometry (Fortessa S10).

## Supplementary information

Supplementary Information

Supplementary Table S1

Supplementary Table S2

Supplementary Table S3

## Data Availability

The RNA-seq raw data generated during and/or analyzed during the current study are available in the Gene Expression Omnibus (GEO-NCBI) repository, accession number: GSE115334.
