# Supplementary material for: Long non-coding RNA FAM83H-AS1 is regulated by human papillomavirus 16 E6 independently of p53 in cervical cancer cells
Source: Sci Rep. 2019 Mar 6;9:3662. doi: 10.1038/s41598-019-40094-8 (PMC6403315; doi:10.1038/s41598-019-40094-8)
Supplement: Supplementary file 1 — Supplementary Information [file 41598_2019_40094_MOESM1_ESM.pdf]

Long non-coding RNA FAM83H-AS1 is regulated by human papillomavirus 16 E6 independently of p53 in cervical cancer cells

Jamie A. Barr[1], Karen E. Hayes[1], Tayvia Brownmiller[1], Abby D. Harold[1], Rajaganapathi Jagannathan[2], Paul R. Lockman[2], Saleem Khan[3], and Ivan Martinez[1,*]

Department of Microbiology, West Virginia University Cancer Institute, School of Medicine, West Virginia University, Morgantown, West Virginia[1]

Department of Basic Pharmaceutical Sciences, Health Sciences Center, School of Pharmacy, West Virginia University, Morgantown, West Virginia[2]

Department of Microbiology and Molecular Genetics, School of Medicine, University of Pittsburgh, Pittsburgh, Pennsylvania[3]

*Correspondence and requests for materials should be addressed to I.M. (email: ivmartinez@hsc.wvu.edu)

Supplementary Methods.

Cell Culture. Primary human foreskin keratinocytes (HEKa) were cultured as described by supplier (EpiLife® Medium supplemented with Human Keratinocyte Growth Supplement (HKGS)). Primary human cervical keratinocytes (HCK) and J2-3T3 mouse fibroblast feeders were cultured as described by Alison McBride's laboratory (NIH, Bethesda, MD)[1]. HCK were maintained in F-media [3:1 F12:DMEM with 5% Fetal Bovine Serum (FBS), 0.4ug/mL Hydrocortisone, 5ug/mL Insulin, 8.4ng/mL Cholera Toxin, 10ng/mL Epidermal Growth Factor, 24ug/mL Adenine, 10U/mL Penicillin, 10ug/mL Streptomycin, 2mM L-Glutamine, Amphotericin B] containing 10uM Y-27632 ROCK Inhibitor (Tocris). HCK cells were co-cultured with J2-3T3 feeder cells rendered mitotically inactive in 8ug/mL Mitomycin C for 3 hours; these growth-arrested feeder cells were replenished every 3-4 days. J2-3T3 stock cells were cultured in Feeder media [Dulbecco's Modified Eagles Medium (DMEM), high glucose, 10% Newborn Calf Serum, 2mM L-Glutamine, 10U/mL Penicillin, 10ug/mL Streptomycin, Amphotericin B]. J2-3T3 feeder cells are sensitive to trypsin; HCK, J2-3T3 stock, and growth-arrested J2-3T3 cells were centrifuged to remove the trypsin when passaged. HPV-16 positive CaSki cells were cultured in Roswell Park Memorial Institute (RPMI)-1640 medium containing 10% FBS, Streptomycin-Penicillin, HEPES Buffer, Amphotericin B. HPV-16 positive W12/20863 and W12/201402 and HPV-31b positive cervical (CIN-612) were co-cultured in E-media (1:1 DMEM:Ham's Nutrient Mix F12 medium supplemented with 5% FBS, 10mM HEPES Buffer, Penicillin-Streptomycin, Amphotericin B, 0.02µM Triiodothyronine, 0.4µg/mL Hydrocortisone, 0.1µg/mL Cholera Toxin, 5µg/mL Transferrin, 180µM Adenine, 5µg/mL Insulin) with murine 3T3 fibroblast cells (3T3M) that were rendered mitotically inactive with 4ug/mL Mitomycin C for at least 2 hours. HPV-negative cervical C33A cells, HPV-18 positive cervical (HeLa), HPV-16 positive HNSCC cell lines UMSCC-47 and -104, HPV negative HNSCC cell line UMSCC-1, HEK293T, and 3T3M cells were all cultured in DMEM supplemented with 10% FBS, Penicillin-Streptomycin, HEPES Buffer, and Amphotericin B. To passage stock cells, 0.25% trypsin was utilized. Growth-arrested J2-3T3 and 3T3M fibroblasts were removed with Versene solution and gently pipetting, prior to pelleting and when otherwise desired. Cells were cultured at 37°C in a humidified 5% CO_2 cell culture incubator.

Generation of stable cell lines. Plasmids MSCV-N-GFP (Plasmid #37855), MSCV-N-16E6 (Plasmid #37875), and MSCV-N-16E7 (Plasmid #37881) were purchased from Addgene. For retroviral production, these plasmids were co-transfected with packaging (pCL-ECO) and envelope (pVSV-GF) plasmids into HEK293T cells using calcium phosphate co-precipitation. 12-16 hours later, HEK293T media was replaced with fresh target cell media. After 48-hour incubation, retrovirus-containing target cell media was harvested, filtered (0.45µm), and mixed with polybrene (4µg/mL final concentration) to increase infection efficiency. 24 hours before infection, co-cultured J2-3T3 fibroblasts were removed with Versene solution and gentle pipetting from HCK cells. HEKa and HCK cells were infected with recombinant retroviruses for 8 hours, virus was removed and replaced with fresh target cell media. Growth arrested J2-3T3 were added to infected HCK and replenished every 3 days. Cells were allowed to recover for 24 hours prior to 72-hour puromycin selection (HEKa-2.5µg/mL; HCK-3µg/mL). Cells were allowed to recover and expression of exogenous HPV-16 E6 and E7, as desired, was confirmed by RT-PCR (Fig. S1, Fig. S3).

Generation of HPV-16 positive cervical JAMM-16 cell line. According to Buck et al. protocol described previously[2], the HPV-16 insert was cut out of pBR322 HPV-16 plasmid by performing a restriction digestion of two separate 25ug plasmid reactions in 225uL each with the restriction enzyme BamHI (NEB) according to manufacturer's recommended conditions (37°C for 2 hours). Digested samples were then PCR purified by QIAquick® PCR Purification Kit (Qiagen) resuspended in 200uL Buffer TE. Purified samples were then ligated under dilute conditions (9mL total volume) by adding 1X Ligase Buffer and 6uL of high concentration (2m U/mL) T4 DNA Ligase (NEB) and incubated at 16°C overnight. <25ug in 9mL ligation reactions were conducted to avoid concatemer formation. The ligated samples were then treated with 4.5mL of 7.5M Ammonium acetate and mixed. 35mL of 95% ethanol was added to each, tubes were mixed, and incubated at 4°C overnight. The next day, the samples were brought back to room temperature and centrifuged at ~5,000 x g at room temperature for 1 hour. Pellets were washed with 70% ethanol, spun briefly, and washed again with 70% ethanol. The pellets were spun one last time to remove any residual ethanol, then air dried for several minutes. Pellets were resuspended in 100uL Buffer TE each, pellets were combined, and Nanodrop was used to calculate concentration retrieved. The entire product was run in 1% agarose gel for 3 hours. Desired band [expect to see supercoiled and relaxed circular bacterial backbone (~1.8 and ~3.2 kb) and supercoiled and relaxed circular (nicked) HPV genome (~6.2 and >16 kb)] was cut out and gel extracted with QIAquick® Gel Extraction Kit (Qiagen). Before transfection of the HPV-16 genomes, growth-arrested J2-3T3 fibroblasts were removed by Versene solution and pipetting from co-cultured primary normal cervical keratinocytes (HCK). HCK were plated in a 6-well plate on day prior to transfecting according to manufacturer's protocol for Lipofectamine LTX (Invitrogen). On day of transfection, HCK were at about 70% confluency. Media was replenished on each well. For each well, 2.8ug of re-ligated HPV-16 insert was diluted in 150uL Opti-MEM I Reduced Serum Media with 2.5uL PLUS Reagent, 15uL Lipofectamine LTX was diluted in Opti-MEM I Reduced Serum Media. Diluted DNA and diluted Lipofectamine LTX/Plus complex were combined and incubated for 5 minutes at room temperature prior to adding to well. The transfected cells were passaged at least 10-15 times so HPV-16 immortalized cells could overgrowth the untransfected HCK cells prior to experimentation.

RNA Extraction. Total RNA of cultured cell lines and human tissues was purified with TRIzol reagent (Invitrogen) and treated with Turbo DNA-free DNase (20 minutes at 37°C, Ambion) according to manufacturers' protocols. RNA concentration was determined with a Nanodrop 2000 Spectrophotometer (Thermo Scientific).

cDNA synthesis, qualitative or quantitative RT-PCR. RNA was reverse transcribed to complementary DNA (cDNA) under standard iScript cDNA synthesis kit (Bio-Rad) instructions in T100^{TM} Thermal Cycler (Bio-Rad). Exogenous spike of *C. Elegans* (70ng) was added for normalization of results if GAPDH mRNA levels were altered during processing (cellular fractionation). RT-PCR or qRT-PCR using SsoAdvanced^{TM} Universal SYBR® Green enzyme was performed according to manufacturer's protocol (Bio-Rad) in CFX Connect^{TM} Real-Time System (Bio-Rad). The oligonucleotide primer sequences can be found in Supplementary Table S3. GAPDH was primarily used as housekeeping gene, however Ubiquitin C (UBC) or Glucuronidase Beta (GUSB) were used if GAPDH mRNA levels were altered with significant apoptosis (siRNA FAM83H-AS1 vs. siRNA-CTRL) and ama-1 was used for cellular fractionation studies. To validate cell fractionation efficiency, U6 snRNA was used as nuclear control and mature mRNA beta-actin as cytoplasmic control. Qualitative RT-PCR product was resolved by agarose gel electrophoresis to visualize or quantitative RT-PCR (qRT-PCR) relative expression was calculated using the double delta CT method (relative expression = 2-ΔCT; where ΔCT=CT (Target RNA) - CT (mRNA endogenous GAPDH/UBC control or *C. Elegans* mRNA Ama-1 was exogenous spike control). Fold changes were calculated relative to control siRNA or the mean value of normal samples.

Western Blot analysis. Cell lysates were boiled in sample buffer, separated by SDS-PAGE, and transferred onto Immobilon-Fl polyvinylidene difluoride membranes. After blocking for 1 hour with 5% milk-TBST buffer (5% non-fat dry milk, 25mM Tris-HCl pH 8.0, 125mM NaCl, 0.5% Tween-20), the membranes were incubated with primary antibodies against human p53 (Cell Signaling, #9282) or Actin (C-11) (Santa Cruz, sc-1615) overnight at 4°C. The membranes were then washed with TBST, incubated with appropriate horseradish peroxidase (HRP)-conjugated secondary antibodies [anti-goat IgG (Santa Cruz, sc-2020); anti-mouse IgG (Thermo Scientific)] for 1 hour at room temperature, washed with TBST, and the proteins were detected on the membrane using Pierce SuperSignal West Pico or Femto Maximum Sensitivity Substrate (Thermo Scientific) chemiluminescence.

Transient transfection. The following siRNAs were used to knock down FAM83H-AS1: Lincode Human FAM83H-AS1 siRNA - Set of 4 (Dharmacon, RU-188909-00-0002), Lincode Human FAM83H-AS1 siRNA SMARTpool (Dharmacon, R-188909-00-000), Lincode Non-targeting siRNA #1 (Dharmacon, D-001320-01-05). Two different siRNA against HPV-16E6: 5'-GAGGUAUAUGACUUUGCUUTT-3' (Dharmacon) and 5'-UCCAUAUGCUGUAUGUGAUTT-3' (siRNA 209, Dharmacon), two different siRNA against p53 (NEB, #2011S; Thermo Scientific Dharmacon®, J-003329-16), two different ONTARGETplus Human siRNA against p300 (Dharmacon, J-003486-11-0002; Dharmacon, J-003486-12-0002), and CTLsiRNA (Ambion, AM16104) were used.

HCK, CaSki, and W12/201402 cells were transiently transfected using standard Lipofectamine® RNAiMAX protocol (siRNA FAM83H-AS1 and siRNA HPV-16E6 #1).W12/201402 and CaSki cells were transfected with reverse transfection protocol (siRNA HPV-16 E6 #2 to improve transfection efficiency. For standard transfection, cells were plated at appropriate confluency to be about 70% confluent for transfection 24 hours post-plating. Growth-arrested J2-3T3 fibroblasts were co-cultured with HCK, and on day of transfection removed with Versene solution and gently pipetting. On day of CaSki, W12/201402, and HCK standard transfection, media was replenished on each well. For each well, Opti-MEM I Reduced Serum Media was used to dilute Lipofectamine RNAiMAX (9uL per well of 6-well), as well as siRNA (40pmol per well of 6-well). Diluted RNAiMAX was combined with diluted siRNA, incubated 5 minutes at room temperature and added to each well. Cells were incubated 48 hours in media containing siRNA prior to harvesting, unless otherwise stated.

Cellular fractionation. Growth arrested 3T3M cells were removed by Versene solution and pipetting the day prior to fractionation for the W12/201402. Cellular fractionation protocol was modified from previous publication[3]. On ice, CaSki (~70% confluent) were washed two times with cold PBS, scraped, pelleted, and resuspended in RSB Buffer (10-1.25mM Tris, pH 7.4; 10-1.25mM NaCl; 3-0.38mM MgCl2), incubated on ice for 5 minutes, centrifuged (1500rpm for 4 minutes at 4°C). RSB Buffer was removed and swollen pellets were resuspended in RSB-G40 Buffer [2.5-0.63mM Tris, pH 7.4; 2.5-0.63mM NaCl; 0.75-0.19mM MgCl2; 2.5-0.63% glycerol; 0.125-0.03% NP-40; 0.125-0.03mM DTT; 100U/mL RNasin Plus RNase Inhibitor (Promega)] in ≥ 4 times volume of pellet. Cells were disrupted with Dounce homogenizer; homogenate was centrifuged (4000 rpm, 4 minutes) to pellet nuclear fraction. The cytoplasmic fraction (supernatant) was collected. The pelleted nuclear fraction was resuspended in RSB-G40 Buffer, 3.3% of sodium deoxycholate and 6.6% of Tween20 were added to the volume of RSB-G40 (10% of final volume was sodium deoxycholate and 10% was Tween 20), samples were tapped to mix, incubated on ice for 5 minutes, and pelleted by centrifugation (7000rpm, 3 minutes). The supernatant was collected and combined with first cytoplasmic fraction, and nuclear pellet was washed two times with RSB-G40 Buffer, and then resuspended in RSB-G40 Buffer. Fractionated cells were Turbo DNase I-treated, according to manufacturer's instructions (NEB), prior to RNA extraction to remove bound DNA and improve recovery of RNA. Total cell (second pellet), cytoplasmic, and nuclear RNA was extracted using TRIzol Reagent (Life Technologies) per manufacturer's instructions (Glycoblue was added during isopropanol precipitation step due to small amounts of RNA recovered from nuclear fraction) and treated with Turbo DNAfree DNase (Ambion) for 20 minutes at 37°C. qRT-PCR analysis was performed using cDNA generated using equal concentration of RNA with exogenous *C. elegans* RNA (70ng) added for normalization.

Cell proliferation assay. Cell proliferation was monitored using Cell Counting Kit - 8 (CCK-8) (Sigma-Aldrich). 24 hours (CaSki) or 48 hours (201402) after transfection with FAM83H-AS1 siRNA, CaSki cells were detached with 0.05% trypsin and W12/201402 cells were detached with 0.25% trypsin. After cell detachment, trypsin was inactivated with trypsin neutralizer (CaSki) or E-media containing serum (201402), pelleted, resuspended in appropriate media containing serum, and plated at desired concentration (3E3 cells per well and of four separate 96-well plates; technical triplicates). In equal density per well, 3T3M fibroblasts treated with mitomycin C were added to the W12/201402 cells and fibroblast alone control wells were plated. At desired time (48, 72, or 96h post-plating), CCK-8 dye (100uL/well) was added to each well, incubated for 2 hours (CaSki) or 3 hours (201402). Then, absorbance was read with a spectrophotometer (BioTek Synergy H1 Hybrid Reader) (OD_{450}) according to manufacturers' protocols. Absorbances of feeders alone were subtracted from W12/201402 co-cultured with feeders. Biological replicate was conducted.

Flow-cytometric analysis of cell cycle. 3T3M fibroblasts were stained with CellTrace™ Far Red Cell Proliferation Kit (Invitrogen) the day before plating transfected 201402 cells. 24 hours (CaSki) or 48 hours (201402) after transfection with FAM83H-AS1 siRNA, cells were detached with 0.05% trypsin (CaSki) or 0.25% trypsin (201402). After cell detachment, trypsin was inactivated with trypsin neutralizer (CaSki) or media containing serum (201402), and cells were plated at desired concentration (4E6 for CaSki and 2.3E6 for 201402) in 100mm dishes in media containing fetal bovine serum (FBS). Mitomycin C treated, far red stained 3T3M cells were added to the 201402 cells. Cells were allowed to attach (about 5 hours) then were washed 3 times with 1X D-PBS to remove residual FBS. Cells were then serum-starved for 24 hours. After incubation, cells were trypsinized with 0.25% trypsin, suspended in serum-free media (to avoid stimulation by serum), washed with 1X PBS, pelleted, and resuspended in PBS (200μL). Harvested cells were then fixed by adding 70% cold ethanol (1.8mL) while vortexing the cells and stored at 4°C until processing. Cells in ethanol were pelleted, washed with PBS, centrifuged, and

resuspended in room temperature 0.2% Tween 20 in PBS (300-1,000µL, depending on pellet size). 100uL of each sample was placed in a U-bottom 96-well plate (3 wells/technical triplicates) and incubated 15 minutes at 37°C. PBS (100uL) was added to each well, cells were pelleted, resuspended in 10uL of RNase A-PBS (180ug/mL stock), tapped gently to mix, and incubated at room temperature for 15 minutes. After incubation, 20uL of PI-PBS (final concentration of 50µg/mL) was added to each well, pipetted to mix, and incubated for 15 minutes at room temperature. After incubation, the volume was brought up to 300uL and analyzed in the dark by flow cytometry (Fortessa S10). Gates were placed around 201402 population. Biological replicate was conducted.

Transwell migration assay. 24 hours (CaSki) or 48 hours (201402) after transfection with FAM83H-AS1 siRNA, cells were detached with 0.05% trypsin. After cell detachment, trypsin was inactivated with trypsin neutralizer, pelleted, resuspended in serum-free media, and cells were plated [1E5 (CaSki) or 0.75E5 (201402), 200uL well, technical duplicates] in upper chambers of 24-well transwell (8mm pore size) with 800uL serum-free media in lower chamber. In equal density per well, 3T3M fibroblasts treated with mitomycin C were added to the W12/201402 cells and fibroblast alone control wells were plated. Cells were then allowed to attach for ~3 hours prior to adding fetal bovine serum (final concentration of 20% for CaSki and 5% for W12/201402) to lower chamber. After 24 hours, media in upper chamber was replenished with appropriate fresh serum free media and lower chamber with appropriate fresh media containing with FBS chemoattractant. 48 hours after cells were plated, lower chamber/underside of transwell and upper chamber of transwell were washed with 25% D-PBS. D-PBS was removed and cells from the top of the upper chamber transwell membrane (non-migrated cells) were wiped away using a cotton swab. The lower chamber/underside of the transwell and upper chamber of transwell were washed again with 25% D-PBS and then the underside of the transwell (migrated cells) was fixed with 4% formaldehyde in 25% D-PBS in the lower chamber for 5 minutes at room temperature. The formaldehyde solution was removed and the lower chamber/underside of transwell and upper chamber of transwell were washed with 25% D-PBS. The cells on the underside of the transwell were then stained using 0.5% crystal violet in 20% ethanol in water. The stain was added to the lower chamber, submerging the transwell for 20 minutes at room temperature. The stain was removed and the lower chamber/underside of the transwell and upper chamber of transwell washed 3 times with 25% D-PBS. The top of the upper chamber transwell membrane was then cleaned with a cotton swab. The transwells were allowed to dry overnight and then the underside of the transwell imaged with an Olympus MVX10 microscope. Migration was quantitated with ImageJ. Feeders alone were subtracted from W12/201402 co-cultured with feeders. Biological replicate was conducted.

Flow-cytometric analysis of apoptosis. 3T3M fibroblasts were stained with CellTrace™ Far Red Cell Proliferation Kit (Invitrogen) the day before plating transfected 201402 cells. 24 hours (CaSki) or 48 hours (201402) after transfection with FAM83H-AS1 siRNA, cells were detached with 0.05% trypsin (CaSki) or 0.25% trypsin (201402). After cell detachment, trypsin was inactivated with trypsin neutralizer (CaSki) or media containing serum (201402), pelleted, resuspended in appropriate media containing serum, and cells were plated in 100mm dishes at desired concentration (1.75E6 for CaSki and 1.1E6 for 201402). At 24, 48, and 72h later post-plating, attached and floating cells were collected. On day of harvest, media containing dead cells was collected, PBS was added to the plates, collected, and added to the media. Attached cells were trypsinized with 0.25% tryspin, cells were pelleted (2 to 5E6) at 4°C, resuspended in PBS, and 100uL of each sample was aliquotted into 3 wells of a 96 well plate (technical triplicates). Cells were pelleted, PBS was removed, Annexin V Binding Buffer (100uL; diluted with autoclaved milliQ water) was added to each tube, Annexin V-FITC (5uL) was added to each tube, and incubated in the dark at room temperature for 10 minutes. After incubation, cold Binding buffer (200uL) was added to each tube, cells were pelleted, washed with cold Binding buffer two times, incubated on

ice for 5 minutes in propidium iodine in Binding buffer (2ug/mL final concentration, 300uL), and analyzed by flow cytometry (Fortessa S10) immediately. Gates were placed around 201402 population. Biological replicate was conducted.

Human cervical tissue specimen. 10 human specimens were obtained from patients under the tissue collection protocol [Prognostic Marker (IRB0406147)] at the University of Pittsburgh. Patient samples were categorized as cervical intraepithelial neoplasia stage 3 (CIN3), invasive cervical cancer (CaCx), or non-cancerous.

TCGA Analysis. The Cancer Genome Atlas (TCGA) (https://cancergenome.nih.gov/) contains patient survival and RNA sequencing data from 196 cervical squamous cell carcinoma and endocervical adenocarcinoma (CESC) human patient samples. TCGA-CESC reads per kilobase million (RPKM) data was extracted, and average RPKM for all patients combined was calculated. TCGA also contains RPKM data from 3 individual non-cancerous cervical samples that were averaged together to compare to CESC RPKM values. The Atlas of non-coding RNA in Cancer (TANRIC) (MD Anderson Cancer Center)[4] that utilizes TCGA-CESC dataset to characterize the expression profiles of long non-coding RNAs (lncRNAs) was used to extract FAM83H-AS1 expression in each of the TCGA-CESC patient samples. Classification and Regression Trees (CART) analysis was used to statistically define high/low lncRNA expression groups. The cut-off z score value was 3 to discern between high and low expression groups. Survival curves were estimated by the Kaplan-Meier method. The log-rank test was used to estimate statistical differences between survival curves. GraphPad software was used to make the survival plots.

Statistics. Student's t-test was utilized to determine mean values differences between groups examined and significance was determined at $p \leq 0.05$ (*) and $p \leq 0.01$ (**). Scale bars represent Standard Deviations (SD).

Supplemental References:

1 Liu, X. *et al.* ROCK inhibitor and feeder cells induce the conditional reprogramming of epithelial cells. *The American journal of pathology* **180**, 599-607, doi:10.1016/j.ajpath.2011.10.036 (2012).

2 Schowalter, R. M., Pastrana, D. V. & Buck, C. B. Glycosaminoglycans and sialylated glycans sequentially facilitate Merkel cell polyomavirus infectious entry. *PLoS pathogens* **7**, e1002161, doi:10.1371/journal.ppat.1002161 (2011).

3 Martinez, I. *et al.* An Exportin-1-dependent microRNA biogenesis pathway during human cell quiescence. *Proceedings of the National Academy of Sciences of the United States of America* **114**, E4961-e4970, doi:10.1073/pnas.1618732114 (2017).

4 Li, J. *et al.* TANRIC: An Interactive Open Platform to Explore the Function of lncRNAs in Cancer. *Cancer research* **75**, 3728-3737, doi:10.1158/0008-5472.can-15-0273 (2015).

Supplementary Figure S1

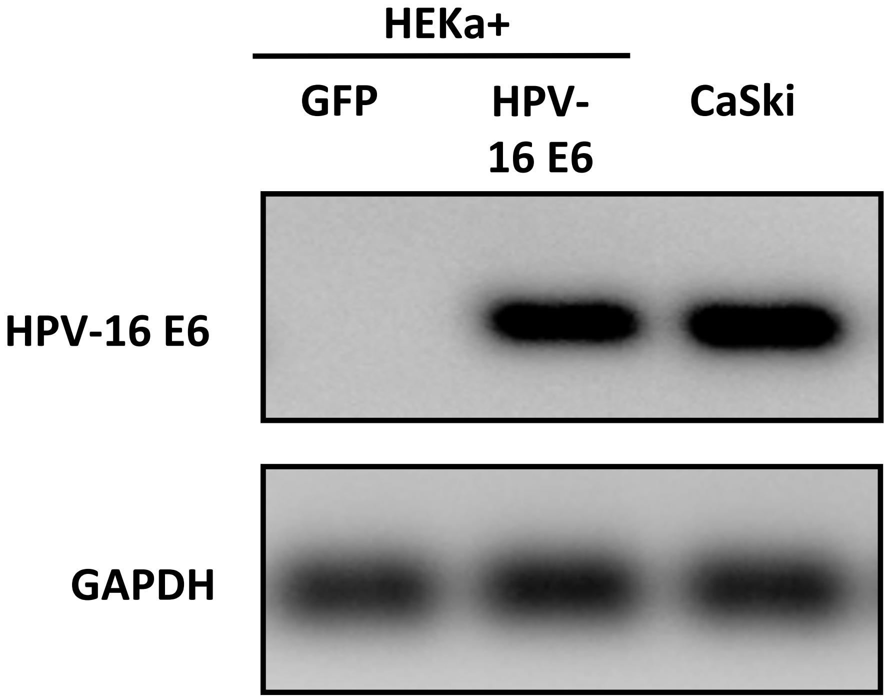

Figure S1: Validation of stable HPV-16 E6 expression in primary foreskin keratinocytes. RT-PCR was used to confirm stable HPV-16 E6 expression in primary foreskin keratinocytes (HEKa) compared to HEKa expressing GFP control and HPV-16 positive CaSki cell line.

Supplementary Figure S2

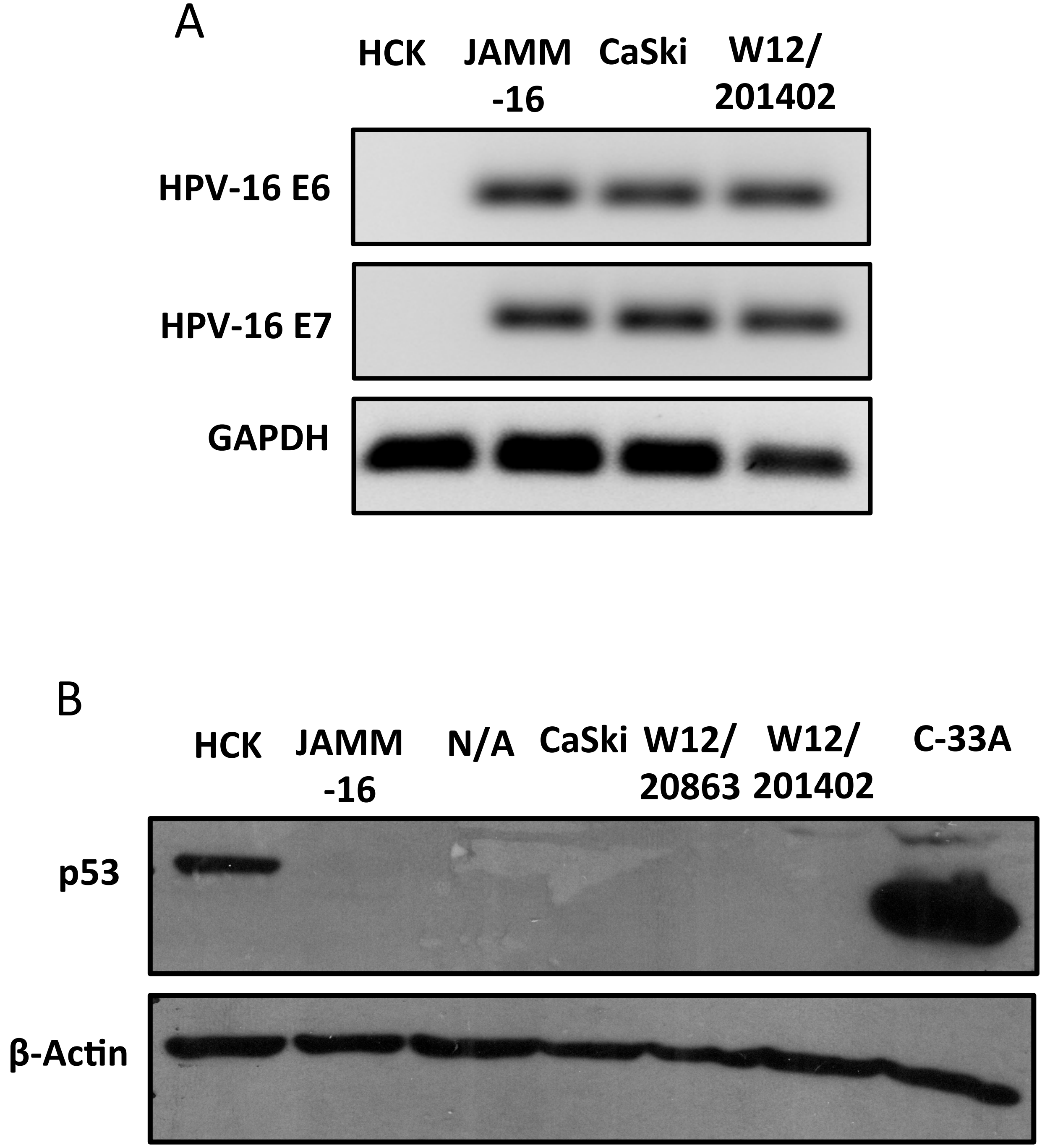

Figure S2: Validation of HPV-16 expression in primary cervical keratinocytes. RT-PCR was used to confirm HPV-16 E6 and E7 expression in cervical keratinocytes transfected with entire HPV-16 genome (JAMM-16) compared to uninfected primary cervical keratinocytes (HCK) and HPV-16 positive CaSki and W12/201402 cell lines. GAPDH was used as loading control. (B) Western blot analysis was used to confirm p53 degradation in HPV-16 positive JAMM-16, CaSki, W12/20863, W12/201402 cell lines compared to HCK and HPV negative, p53-mutated C-33A cell line. Note: N/A represents a cervical cell line that was not used in this study.

Supplementary Figure S3

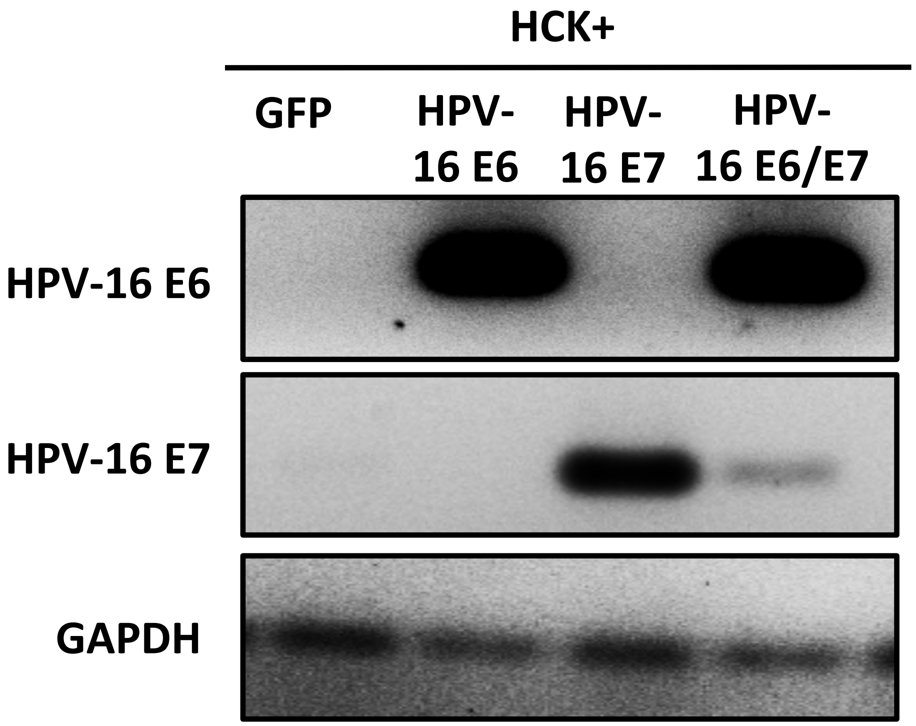

Figure S3: Validation of stable HPV-16 E6 and E7 expression in primary cervical keratinocytes. RT-PCR was used to confirm stable HPV-16 E6 and E7 expression, as desired, in primary cervical keratinocytes (HCK) compared to HCK expressing GFP control.

Supplementary Figure S4

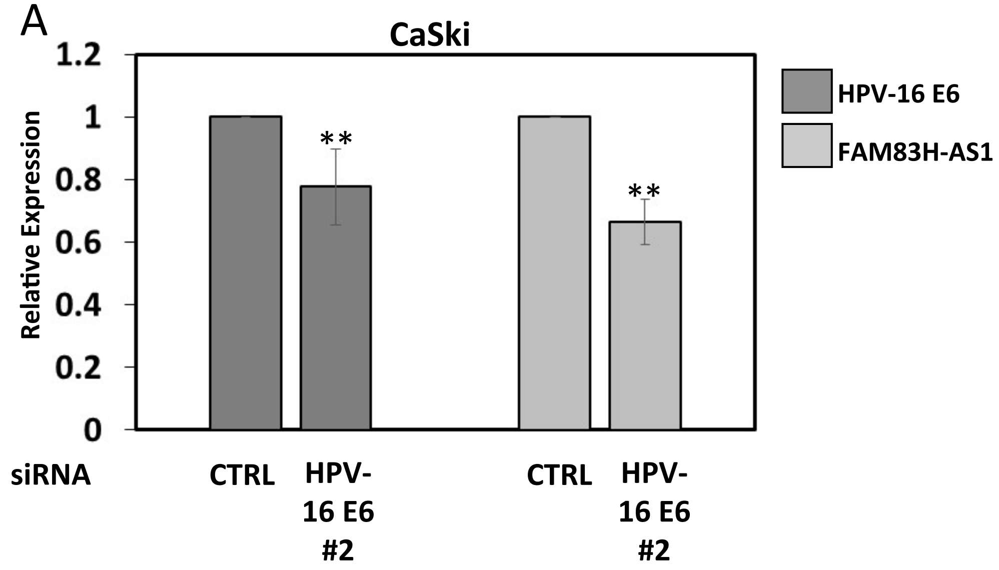

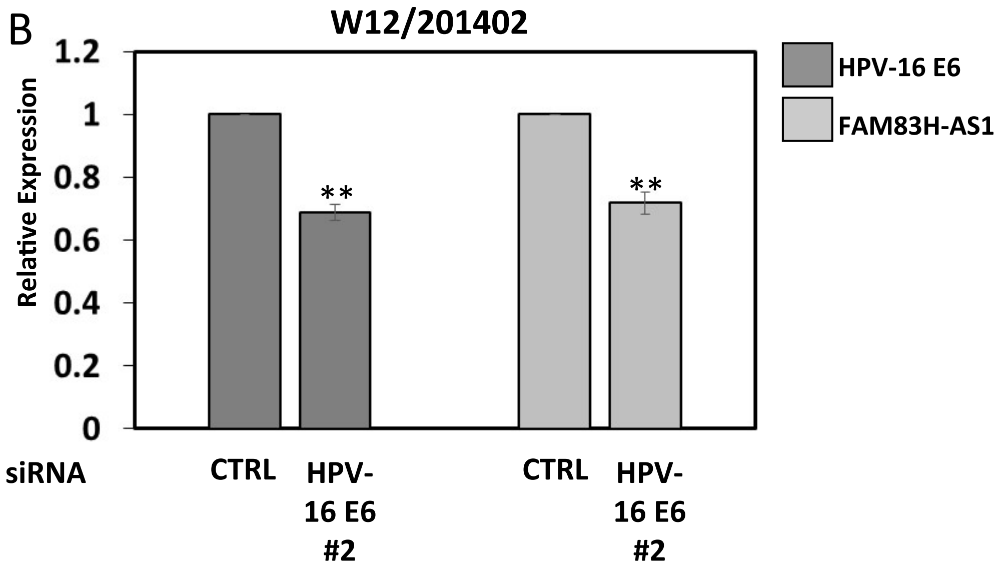

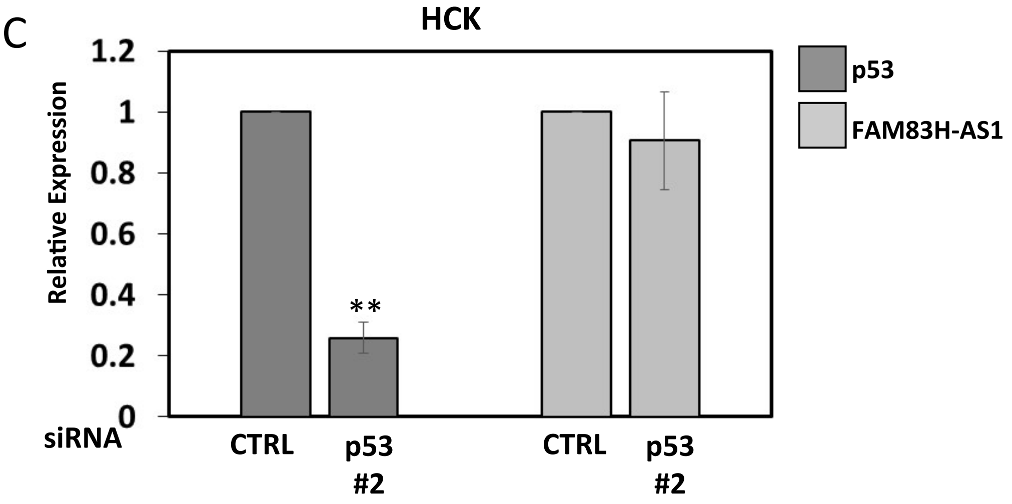

Supplementary Figure S4

D

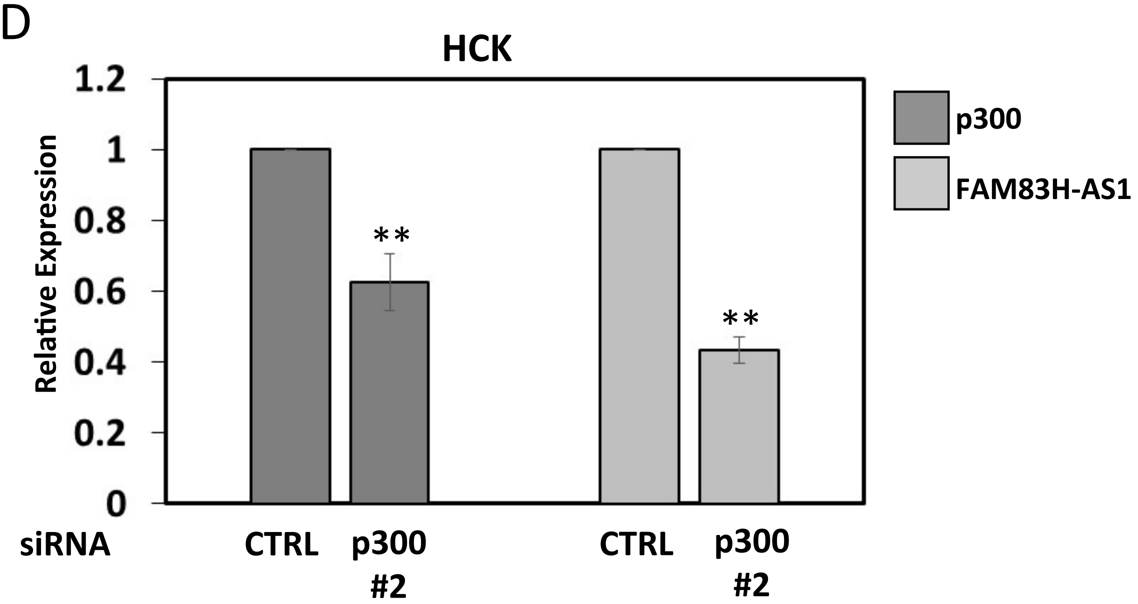

E

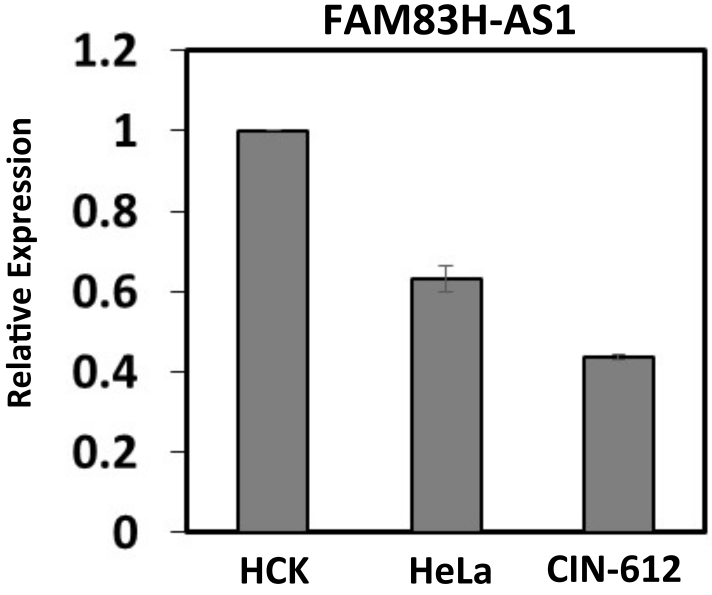

Figure S4: FAM83H-AS1 expression is regulated by HPV-16 E6 in a p53-independent, p300-dependent manner. (A-B) qRT-PCR analysis of HPV-16 E6 and FAM83H-AS1 expression in HPV-16 positive Caski (A) and W12/201402 (B) cell lines transfected with a second siRNA (siRNA #2) against HPV-16 E6 compared to siRNA control. (C) qRT-PCR analysis of p53 and FAM83H-AS1 expression in primary cervical keratinocytes (HCK) transfected with a different siRNA (siRNA #2) against p53 compared to siRNA control. (D) p300 and FAM83H-AS1 expression in HCK transfected with a different siRNA (siRNA #2) against p300 compared to siRNA control. (E) qRT-PCR analysis of FAM83H-AS1 expression in HPV-18 positive HeLa cells and HPV-31b positive CIN-612 cervical cell lines compared to primary cervical keratinocytes (HCK). (A-D) The graphs show the average of two independent experiments. Similar results were obtained in at least three independent experiments. GAPDH mRNA was used to normalize the qRT-PCR analyses. Two-tailed t test results are indicted as ** $p \leq 0.01$. CTRL, control.

Supplementary Figure S5

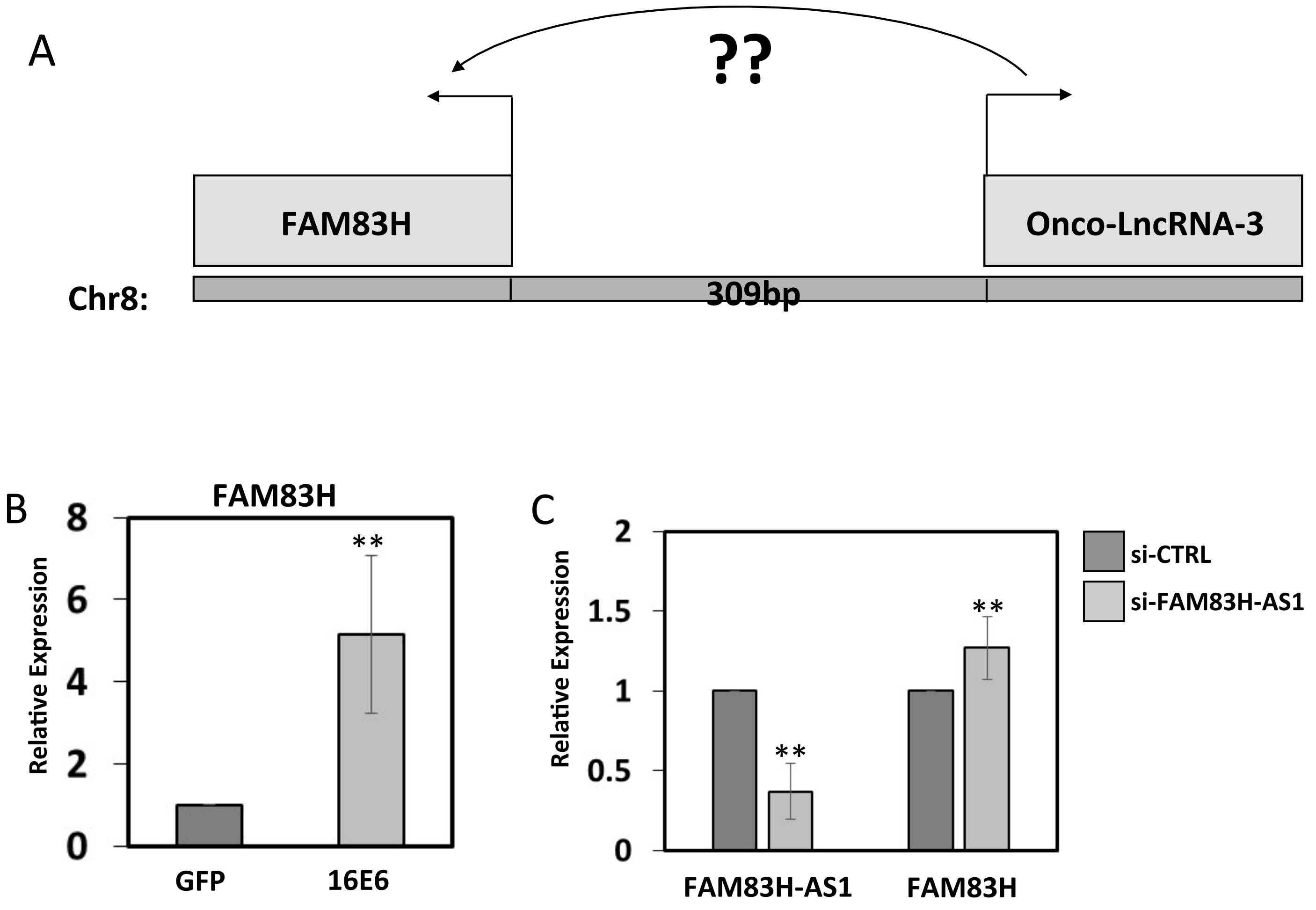

Figure S5: FAM83H expression is not regulated by FAM83H-AS1. (A) Schematic diagram depicting possible cis-regulation of protein coding gene FAM83H by lncRNA FAM83H-AS1. (B) qRT-PCR analysis of FAM83H protein coding gene expression in primary cervical keratinocytes (HCK) stably expressing HPV-16 E6 compared to GFP control. (C) qRT-PCR analysis of FAM83H and FAM83H-AS1 expression in CaSki transfected with siRNA against FAM83H-AS1. The graphs shows the average of two independent experiments. Similar results were obtained in at least three independent experiments. GAPDH mRNA was used to normalize the qRT-PCR analyses. Two-tailed t test results are indicted as ** $p \leq 0.01$. CTRL, control.

Supplementary Figure S6

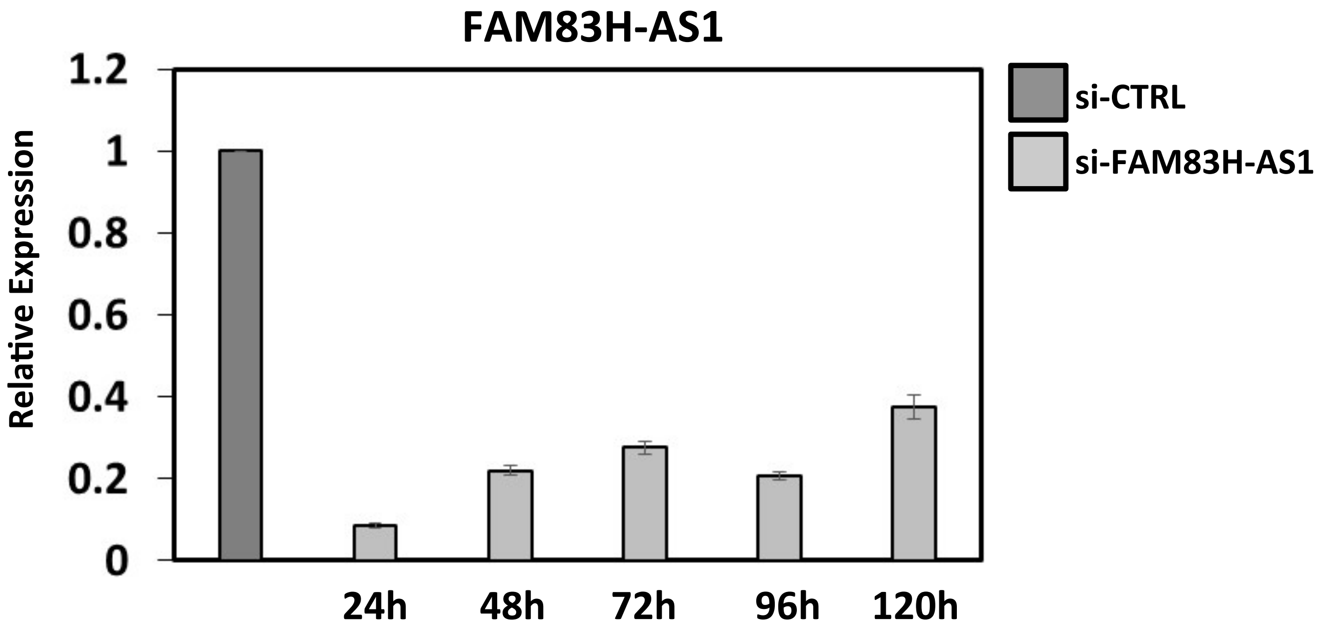

Figure S6: FAM83H-AS1 expression at 24, 48, 72, 96, and 120 hours post-transfection with FAM83H-AS1 siRNA SMARTpool by qRT-PCR analysis.

Supplementary Figure S7

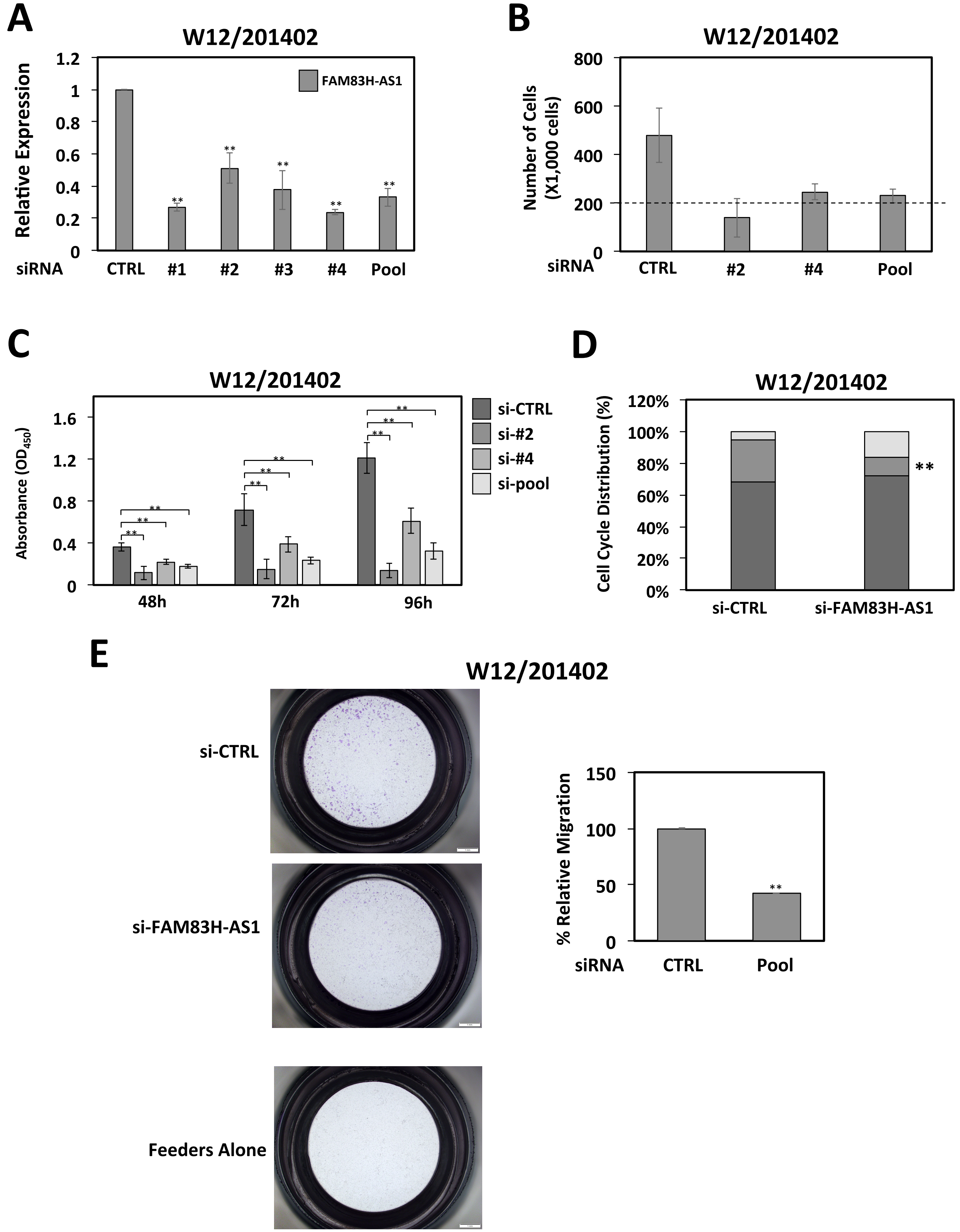

Supplementary Figure S7

F

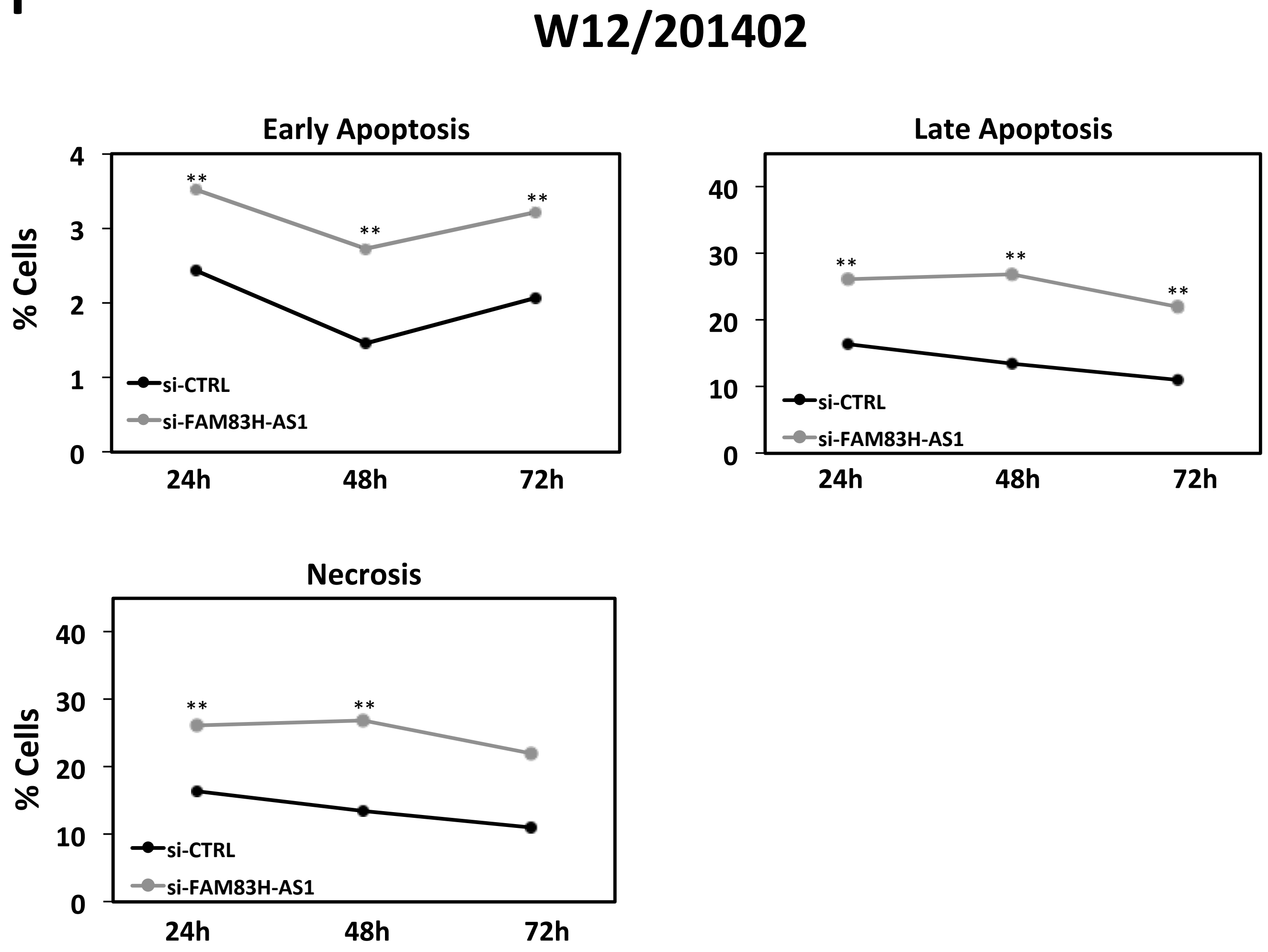

Supplementary Figure S7

G

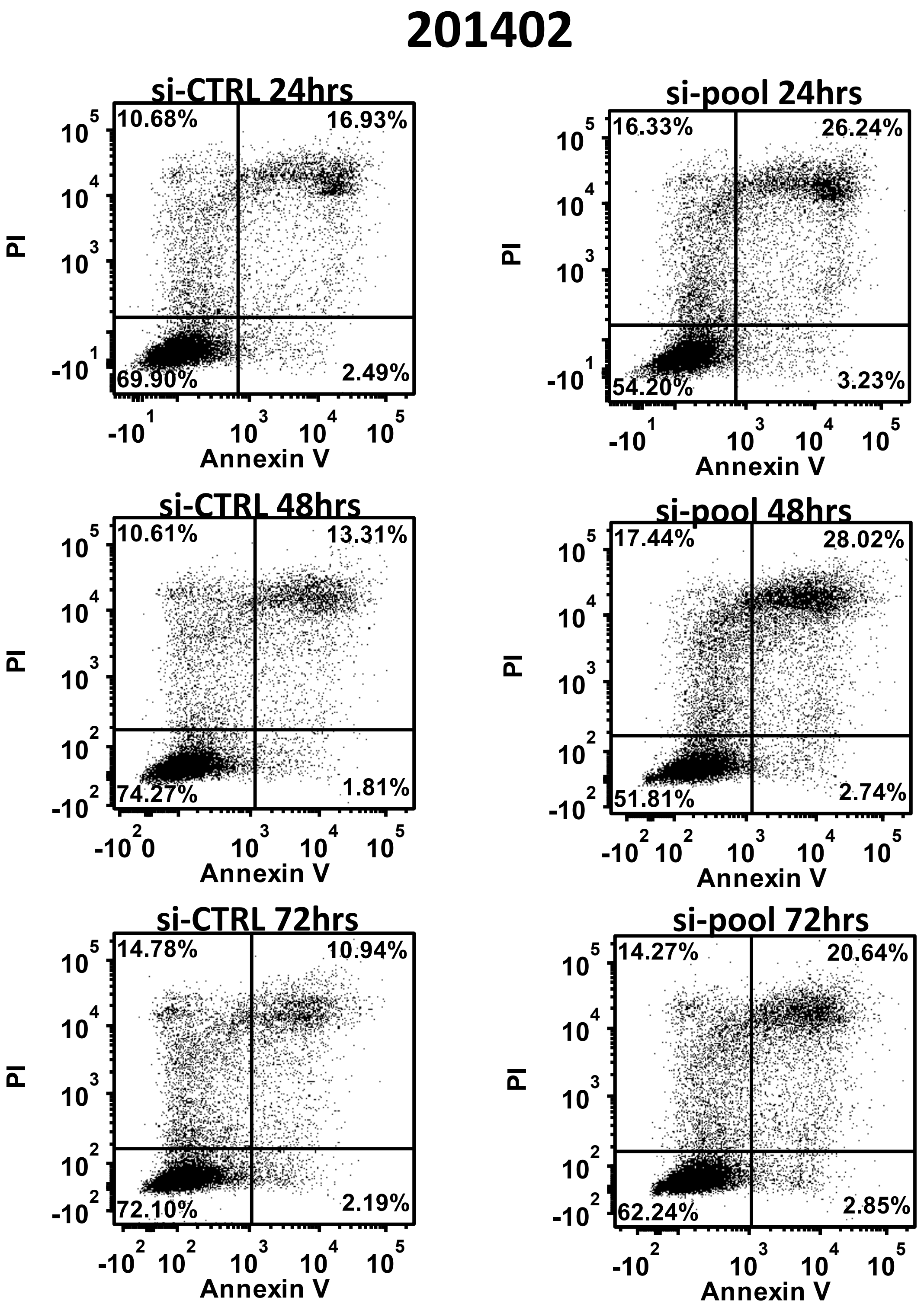

Supplementary Figure S7

H

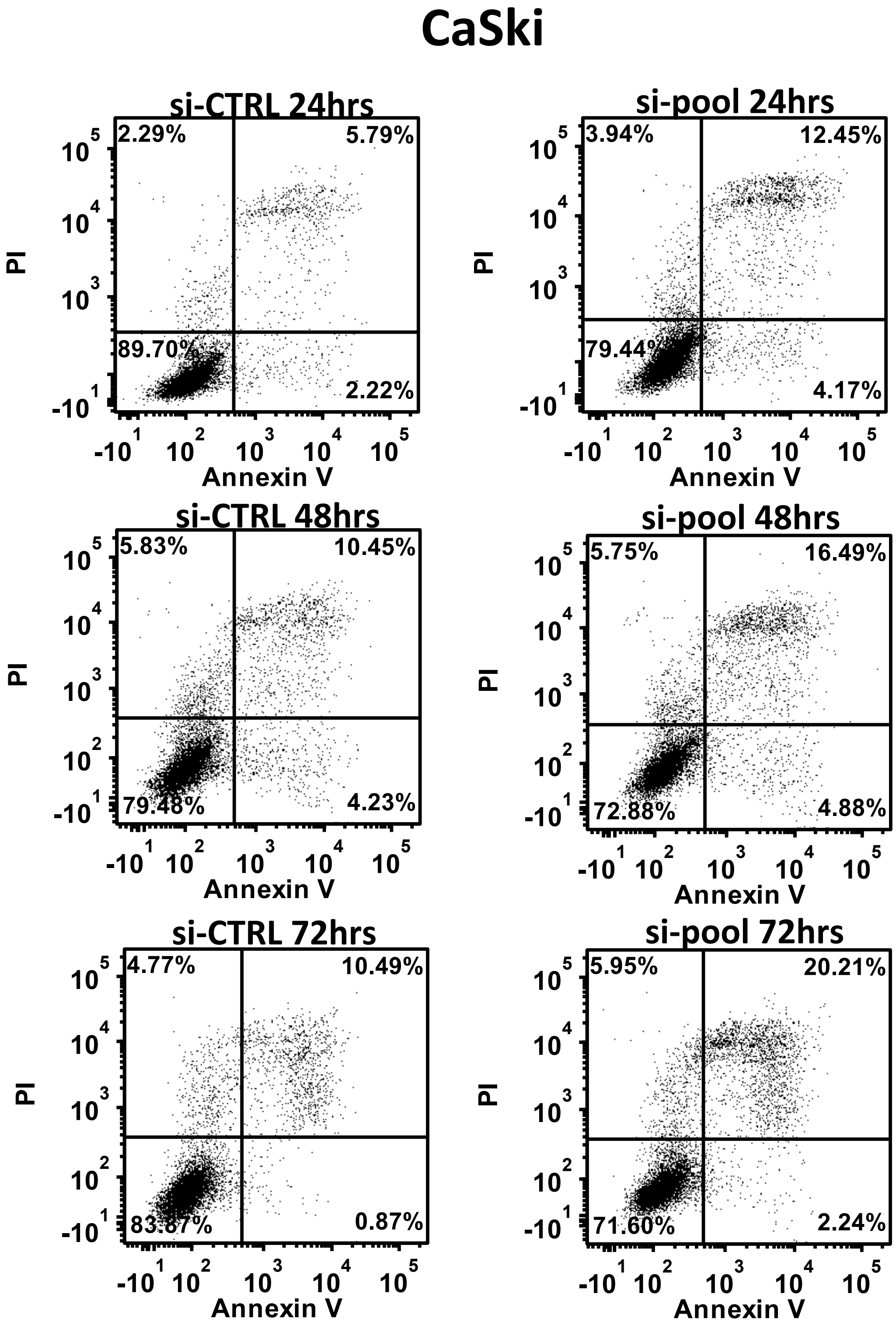

Figure S7: FAM83H-AS1 knockdown altered cell proliferation, migration, and apoptosis in W12/201402 cells. (A) Knockdown efficiency of individual and SMARTpool siRNA against FAM83H-AS1 in HPV-16 positive W12/201402, measured by qRT-PCR analysis. Because of variations in the expression of GAPDH after the knockdown of FAM83H-AS1, we used GUSB mRNA to normalize the qRT-PCR analyses. The graph shows average of two individual experiments. (B) W12/201402 cells were transfected with individual siRNAs against FAM83H-AS1, siRNA SMARTpool against FAM83H-AS1, or siRNA control for 48 hours. Cells were then re-plated in equal numbers (200,000 cells/well, represented by dashed line in graph) and cultured another 48 hours prior to re-counting attached cells. The graph shows the average of two individual experiments. (C) W12/201402 cells were transfected with individual siRNAs against FAM83H-AS1, siRNA SMARTpool against FAM83H-AS1, or siRNA control for 48 hours then plated in equal numbers. Transfected cells were analyzed for cellular proliferation assessment by CCK-8 assay at 48, 72, and 96 hours post-plating. The graph shows the average of two individual experiments. (D-G) W12/201402 cells were transfected with siRNA SMARTpool against FAM83H-AS1 or siRNA control for 24 hours then plated in equal numbers for experiments. (D) Transfected cells were analyzed for cell cycle alterations by FACS analysis. W12/201402 cells with knockdown of FAM83H-AS1 exhibit less cells in S-phase of cell cycle compared to control cells. The graph shows the average of two individual experiments. (E) Transwell migration of transfected cells was analyzed 48 hours post-plating in upper chamber with chemoattractant in lower chamber. The graph shows the average of two individual experiments. (F-H) Transfected W12/201402 or CaSki cells were collected at 1, 2, and 3 days post-plating, stained with Annexin V/PI, and analyzed by flow cytometry to show alterations in apoptosis compared to siRNA control. (F) Quantitative analysis of Annexin V/PI flow cytometry data in W12/201402 cells. The graph shows the average of two individual experiments. (G) Dot plot analysis of Annexin V/PI flow cytometry data in W12/201402 cells. (H) Dot plot analysis of Annexin V/PI flow cytometry data in CaSki cells. Representative images shown for siRNA FAM83H-AS1 and siRNA control at each time point. Two-tailed t test results are indicted as * $p \leq 0.05$ and ** $p \leq 0.01$.
